# Inhibition of insulin-like growth factor II (IGF-II)-dependent cell growth by multidentate pentamannosyl 6-phosphate-based ligands targeting the mannose 6-phosphate/IGF-II receptor

**DOI:** 10.18632/oncotarget.11493

**Published:** 2016-08-22

**Authors:** Megan E. Zavorka, Christopher M. Connelly, Rosslyn Grosely, Richard G. MacDonald

**Affiliations:** ^1^ Department of Biochemistry and Molecular Biology, University of Nebraska Medical Center, Omaha, Nebraska 68198, USA

**Keywords:** biochemistry, insulin-like growth factors, apoptosis, receptor

## Abstract

The mannose 6-phosphate/insulin-like growth factor II receptor (M6P/IGF2R) binds M6P-capped ligands and IGF-II at different binding sites within the ectodomain and mediates ligand internalization and trafficking to the lysosome. Multivalent M6P-based ligands can cross-bridge the M6P/IGF2R, which increases the rate of receptor internalization, permitting IGF-II binding as a passenger ligand and subsequent trafficking to the lysosome, where the IGF-II is degraded. This unique feature of the receptor may be exploited to design novel therapeutic agents against IGF-II-dependent cancers that will lead to decreased bioavailable IGF-II within the tumor microenvironment. We have designed a panel of M6P-based ligands that bind to the M6P/IGF2R with high affinity in a bivalent manner and cause decreased cell viability. We present evidence that our ligands bind through the M6P-binding sites of the receptor and facilitate internalization and degradation of IGF-II from conditioned medium to mediate this cellular response. To our knowledge, this is the first panel of synthetic bivalent ligands for the M6P/IGF2R that can take advantage of the ligand-receptor interactions of the M6P/IGF2R to provide proof-of-principle evidence for the feasibility of novel chemotherapeutic agents that decrease IGF-II-dependent growth of cancer cells.

## INTRODUCTION

The mannose 6-phosphate/insulin-like growth factor II receptor (M6P/IGF2R) is a multifunctional type-1 transmembrane glycoprotein that binds a diverse array of ligands to help regulate multiple cellular functions, including lysosome biogenesis, growth, survival and development [1–3]. This 300-kDa receptor consists of a 2264-residue extracellular domain (ectodomain), a 23-residue transmembrane domain, and a short (164 residues) carboxyl-terminal cytoplasmic domain [4, 5]. The ectodomain consists of 15 homologous (14-28% sequence identity) repeats about 147 amino acids in length, with each repeat forming anti-parallel β-sheets supported by 3-4 disulfide bonds and resembling a flattened β-barrel structure [4–9]. The M6P/IGF2R binds two major classes of ligands: M6P-glycosylated (bearing M6P-moieties; e.g., lysosomal enzymes) and non-glycosylated ligands (i.e., IGF-II). Ligand binding studies and mutational analyses have identified critical residues within the M6P-binding repeats 3, 5, 9, and 15. Although these binding sites are highly homologous, their binding properties differ in preferences for M6P mono- vs. diester compounds, inter-repeat interactions required for functional binding, and pH optima. Repeats 3 and 9 bind M6P-monoesters with high affinity and mediate the majority of lysosomal enzyme binding and trafficking. Repeat 5 binds M6P-monoesters with lower affinity, preferring M6P-diesters. Repeat 15 was recently shown to bind M6P-monoesters with very low affinity, with a *K*_D_ = 13 μM compared to *K*_D_ = ~1 nM for repeats 3 and 9 [8, 10–16]. IGF-II binds to repeat 11 in cooperation with the fibronectin-like domain of repeat 13, which enhances the receptor's affinity for IGF-II [17, 18]. IGF-II binding and M6P-tagged ligand interactions with the receptor are not mutually exclusive, which may allow the M6P/IGF2R to function as a tumor suppressor. It is proposed that extracellular IGF-II is trafficked to the lysosome by the receptor and degraded, thus reducing IGF-II bioavailability for binding the IGF-I receptor (IGF1R) [19, 20]. Internalization of the M6P/IGF2R from the plasma membrane is constitutive, mediating recovery/re-uptake of extracellular M6P-tagged ligands and the uptake of exogenous IGF-II [21–24]. M6P/IGF2R mainly functions to transport newly synthesized acid hydrolases from the *trans*-Golgi network to the lysosomes, where they are lysosome-resident enzymes involved in the degradation of cellular macromolecules [1, 25]. Approximately 10% of the M6P/IGF2R exists on the plasma membrane, where it functions in binding extracellular ligands (M6P-bearing and IGF-II) and internalizing and trafficking them to the lysosome [13].

High-affinity binding of M6P-bearing ligands to the M6P/IGF2R requires bivalent interaction in the form of simultaneous binding of ligands bearing two oligosaccharides capped by a M6P moiety or a single oligosaccharide bearing two M6P groups [26, 27]. York *et al.* observed that β-glucuronidase (hGUS), a homotetrameric lysosomal enzyme bearing multiple M6P groups, increased the rate of internalization of IGF-II bound to the M6P/IGF2R by cross-bridging the M6P binding sites on two subunits of the receptor dimer by 3- to 4-fold [28]. Neither the monovalent ligand M6P nor IGF-II itself was able to produce the same response, suggesting that they were not capable of cross-bridging the receptor into a dimeric structure. Moreover, cellular repressor of E1A-stimulated genes (CREG), a secreted M6P-capped glycoprotein, can cause internalization of IGF-II that is dependent on M6P/IGF2R, leading to delays in cell cycle progression in human embryonic carcinoma (NTERA-2), smooth muscle cells, and NIH3T3 fibroblast cell lines [29–31]. In summary, these studies suggest that binding of a multivalent M6P-bearing ligand to the M6P/IGF2R can enhance the receptor's internalization of IGF-II. We propose that this mechanism may be leveraged for the treatment of cancers by exploiting the M6P/IGF2R-mediated destruction of IGF-II to inhibit progression of IGF-II-dependent tumors.

The present study aimed to test the hypothesis that the M6P/IGF2R can be targeted by a panel of bidentate and multidentate M6P-based ligands that stabilize the dimeric structure of the receptor and promote internalization of pericellular IGF-II, leading to reduced IGF-II-dependent cell growth. Therefore, as proof-of-principle to test this hypothesis, we synthesized a panel of bi- and multidentate pentamannosyl 6-phosphate (PMP)-based pseudoglycoproteins and glycopeptides of different molecular sizes, that could be used to identify the smallest M6P-based ligand that would achieve high-affinity, bivalent binding to the M6P/IGF2R. Radioligand displacement assays indicate that, when compared to the low-affinity, monovalent ligand M6P, all these compounds bind to the M6P/IGF2R with high affinity, indicative of a bivalent binding mechanism. Cell growth studies suggest that these compounds are capable of decreasing viability in several IGF-dependent cancer cell lines. IGF-II internalization/degradation assays demonstrated that incubation of cells with the PMP-based ligand promoted uptake and degradation of IGF-II.

## RESULTS AND DISCUSSION

### Design, synthesis and purification of pentamannosyl 6-phosphate (PMP)-derivatized proteins and peptides

Previously, we have evaluated several panels of synthetic, bidentate M6P-based compounds that we found were low-affinity ligands for the M6P/IGF2R [32, 33]. Their low affinity was attributed to the possibility that the phosphate-to-phosphate end distance of these compounds was not able to span the molecular distance (~30 Å) needed to access two M6P-binding sites of the M6P/IGF2R dimer simultaneously. Therefore for the current study, we synthesized a panel of ligands based on protein scaffolds varying in molecular size to determine the minimal size needed to achieve high-affinity binding to cross-bridge the receptor. Pentamannosyl 6-phosphate (PMP) derived from a yeast phosphomannan was coupled by reductive amination to protein scaffolds of different sizes, including albumin (PMP-BSA), ovalbumin (PMP-OVA), and insulin (PMP-INS). We have also chemically linked PMP to two tripeptides: lysyl-tyrosyl-lysine (PMP-KYK) and seryl-tyrosyl-lysine (PMP-SYK). The PMP-pseudoglycoproteins were purified by dialysis and analyzed by SDS-PAGE; Coomassie staining of the gels revealed purified products that shifted to molecular masses indicative of a high percentage of derivatization of PMP to BSA, OVA and INS (Table 1). The PMP-pseudoglycopeptides were purified by anion-exchange and size-exclusion chromatography; analysis by MALDI-TOF mass spectrometry suggested that the PMP-glycopeptides were heterogeneous in size, with mass differences corresponding to differences in length of the oligomannose chains (data not shown).

**Table 1:** Molecular Characteristics and Binding Properties of the PMP-peptide and PMP-protein Ligands for the M6P/IGF2R

PMP-Ligand	Precursor	Predicted MW	Approximate PMP Groups	IC_50_, nM^a^	RBA^b^
PMP-BSA	Bovine Serum Albumin	90,000	24	17 ± 5 (3)	4240
PMP-OVA	Chicken Egg Ovalbumin	58,000	13	21 ± 4 (3)	3430
PMP-INS	Bovine Insulin	8,800	3	13 ± 1 (4)	5540
PMP-KYK	Lysyl-Tyrosyl-Lysine	3,580	3	120 ± 3 (3)	600
PMP-SYK	Seryl-Tyrosyl-Lysine	2,378	2	52 ± 1 (3)	1380

**Figure 1:**
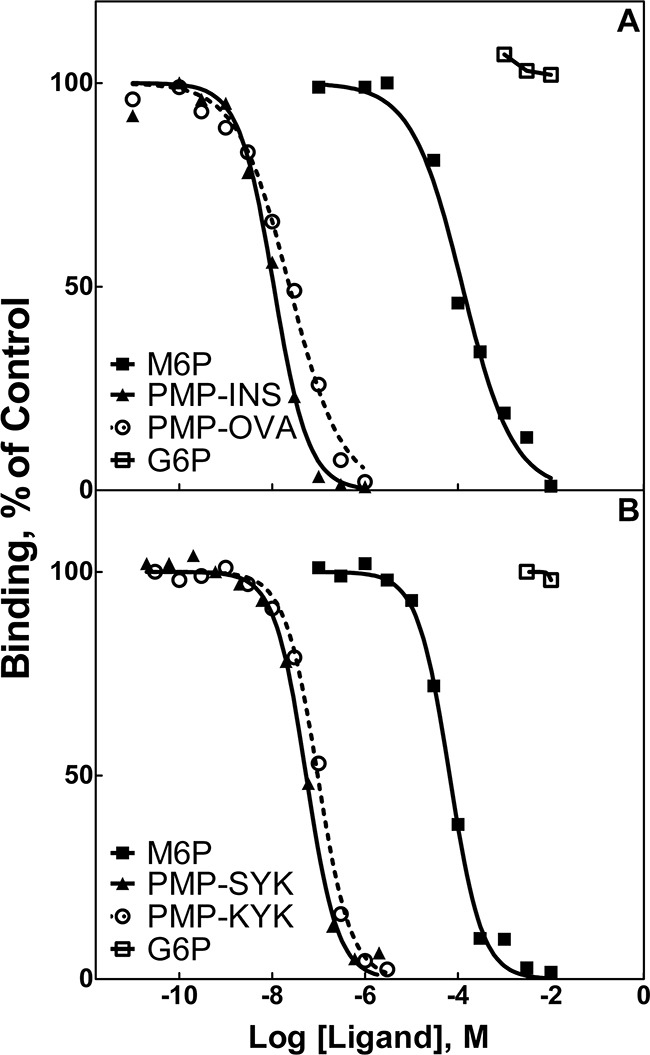
Competitive binding analysis for PMP-INS, PMP-OVA and PMP-peptides in displacement of radiolabeled PMP-BSA from M6P/IGF2R-Sepharose Increasing concentrations of the test compounds were incubated with the tracer radioligand, ^125^I-PMP-BSA (5 × 10^5^ cpm), with 10 μL aliquots of soluble bovine M6P/IGF2R-Sepharose resin in 0.2 mL volume of HBS plus 0.05% Triton X-100 for 16 h at 4°C. Radioactivity associated with the washed pellets was measured in a gamma counter. Best-fit curves for the data were obtained by Prism GraphPad™ software for calculation of the concentration of each compound required to displace 50% of the tracer (IC_50_). M6P and G6P were analyzed in parallel as positive and negative controls, respectively. Data for: A. PMP-derivatized proteins, PMP-INS and PMP-OVA. B. PMP-derivatized peptides, PMP-Ser-Tyr-Lys (PMP-S-Y-K) and PMP-Lys-Tyr-Lys (PMP-K-Y-K).

The panel of PMP-based ligands was designed to have various MW and different stoichiometry of PMP groups per ligand, but all produced bivalent binding for the M6P/IGF2R. The precursor protein or peptide, predicted molecular weight following conjugation, and approximate number of conjugated PMP groups are listed for each ligand.

a IC_50_ values for competitive displacement of radiolabeled PMP-BSA from the receptor calculated from multiple experiments (n = # of trials) such as that shown in Figure 1.

b RBA = relative binding affinity, normalized to free M6P.

### M6P/IGF2R-binding properties of the PMP-based ligands

To measure binding of these ligands to the M6P/IGF2R, radioligand displacement assays were performed using a series of ligand concentrations with either ^125^I-hGUS or ^125^I-PMP-BSA as tracers. Soluble M6P/IGF2R, purified from fetal bovine serum and coupled to Sepharose 4B resin, served as the receptor source (Figure 1). The IC_50_ values calculated for PMP-OVA, -INS, and -BSA (Table 1) indicated that all three ligands are of high affinity relative to the monovalent binding of M6P (*K*_D_=8 μM) [26, 32, 33]. The IC_50_ data for the PMP-glycopeptides indicated a slightly lower affinity than the PMP-glycoproteins, but these values were still considered to be high-affinity, with estimated *K*_D_ values in the nanomolar range (Table 1). The lower values of relative binding affinities (RBAs) for the PMP-peptides relative to the PMP-proteins may be due to steric issues resulting from the higher conformational flexibility within the peptide tether, which would allow the PMP to sample multiple conformations in solution. In contrast, the PMP-proteins have a more defined tertiary structure of the scaffold with more stable placement of the PMP groups, which would constrain the conformational space that the PMP groups can sample. Additionally, the PMP-proteins have more M6P groups than the PMP-peptides, allowing for the ligand to find a more optimal geometry for interaction with the receptor. This also may partially explain why hGUS, a natural ligand with multiple high-mannose *N*-linked oligosaccharides, possesses a lower IC_50_ and higher RBA than our PMP-ligands. Thus, the stable positioning and presence of multiple high-mannose oligosaccharides capped with M6P on hGUS are important features contributing to its high affinity, and these attributes may optimize its ability to promote dimerization and rapid internalization of the M6P/IGF2R. These data support the idea that small molecules with end-to-end M6P moieties with estimated distance of 60-70 Å can bind the M6P/IGF2R in a bivalent, high-affinity manner.

### Binding mechanism of the PMP-based ligands

Previous work has demonstrated that the natural ligand, hGUS, enhanced internalization by binding to the M6P/IGF2R in a 2:1 receptor-to-ligand stoichiometry, suggesting that this multidentate ligand bound to M6P-binding sites on separate M6P/IGF2R monomers rather than via intramolecular interaction with the sites in repeats 3 and 9 [28]. Additionally, Byrd *et al.* demonstrated that the pseudoglycoprotein, PMP-BSA, preferred to bind pre-formed receptor dimers over the monomeric M6P/IGF2R [27]. The bivalent binding mechanism should result in a 100- to 1000-fold increase in binding affinity for the M6P/IGF2R relative to monovalent ligands, such as M6P or PMP.

**Figure 2:**
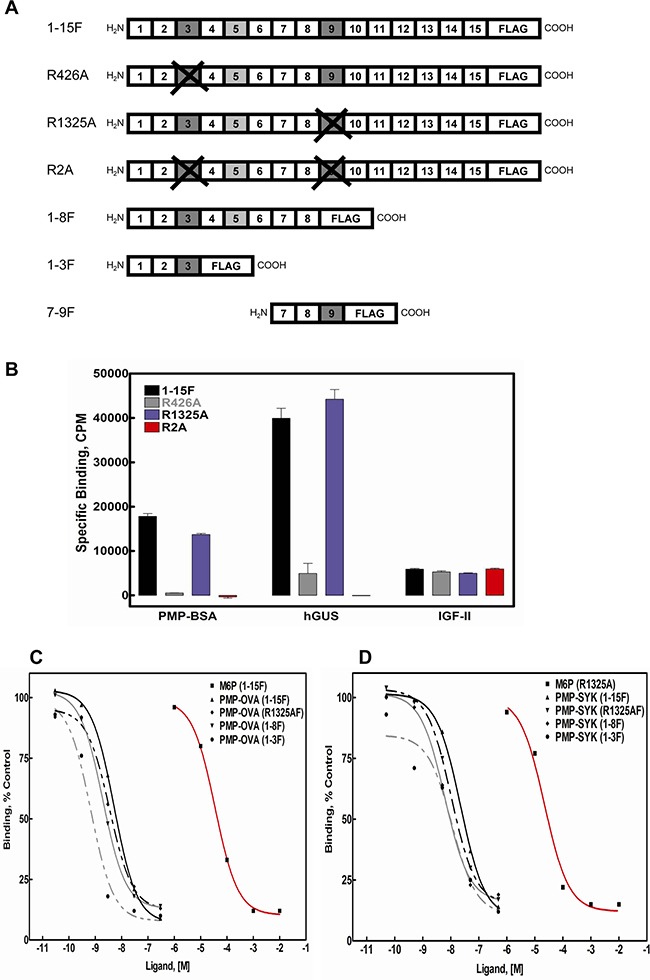
Competitive binding analysis for PMP-OVA and PMP-SYK in displacement of PMP-BSA from M6P-IGF2R-FLAG constructs immobilized on M2-α-FLAG affinity resin Cell lysates, containing equimolar amounts of the indicated expressed soluble receptors **A.** were immunoprecipitated with M2 α-FLAG affinity resin and tested for direct binding to ^125^I-PMP-BSA, ^125^I-hGUS, or ^125^I-IGF-II **B.**, or incubated with increasing concentrations of PMP-OVA **C.**, or PMP-SYK **D.** to measure the ability of the PMP-ligands to displace the radiolabeled tracer, ^125^I-PMP-BSA (5 × 10^5^ cpm). Data were analyzed as described in the legend to Figure 1. Data for M6P and G6P were analyzed in parallel as positive and negative controls, respectively. The curves for M6P for each expressed soluble receptor were measured and represented as a displacement curves for a single immunoprecipitated soluble receptor. G6P had no apparent displacement activity for any of the test ligands, and these curves have been omitted for clarity.

To test this property for our panel of PMP-based ligands, we utilized M6P/IGF2R mini-receptors designed to include all or some of the 15 ectodomain repeats followed by a C-terminal FLAG epitope tag (Figure 2A). In addition to the wild-type, full-length ectodomain construct (1-15F), some full-length constructs contained arginine-to-alanine substitutions within the 3^rd^ (R426A) or 9^th^ (R1325A) domains that abolish M6P-based binding of those sites [34], while the truncated mini-receptors bear deletions that eliminate entire M6P-binding domains. By regulating the number of available M6P-binding sites per receptor, we should be able to assess whether the PMP-based ligands achieve high affinity binding to the receptor by interaction with multiple sites on a single monomer or by cross-bridging sites on a dimeric receptor. In the latter case, we would expect that the IC_50_ values using M6P/IGF2R constructs with a single high-affinity M6P-binding site per monomer would be similar to the values for wild-type receptor obtained in Figure 1. The FLAG epitope-tagged constructs were expressed in HEK 293 cells, and Triton X-100 cell extracts and conditioned media were analyzed for relative expression levels of the mini-receptors by immunoblotting with M2 α-FLAG antibody, revealing expression of all constructs (data not shown).

To determine if PMP-based ligands had a preference for binding to the M6P-binding sites in domains 3 or 9, equimolar amounts of the FLAG-receptor constructs were immunoadsorbed to M2 α-FLAG resin and we compared the direct binding of ^125^I-PMP-BSA to the wild-type M6P/IGF2R construct or the various mutants. The data demonstrate that hGUS and PMP-BSA have a distinct preference for the M6P-binding site located in repeat 3 of the receptor (Figure 2B) over that in repeat 9; the R1325A mutation in repeat 9 has minimal effect on binding by either ligand, while the R426A mutation in repeat 3 completely eliminated PMP-BSA binding. However, it is noteworthy that hGUS retains some binding to the R426A mutant construct, suggesting this ligand has some affinity for the repeat 9 site as well as the repeat 3 site. In contrast, the double mutant, R2A, showed no detectable binding with either ligand in this assay. Altogether, these data suggest that most of the M6P-based binding, for both hGUS and PMP-BSA, to the M6P/IGF2R can be attributed to contacts made within the M6P-binding site located within domain 3 of the receptor's ectodomain.

High-affinity binding of M6P-based ligands to the M6P/IGF2R has been shown to arise from intermolecular cross-bridging of two M6P/IGF2R monomers by bivalent ligands [26, 27]. To determine if this is also the case for the PMP-ligands, we tested their binding to a series of mini-receptors having only one high-affinity M6P-binding site, in repeat 3, per monomer. PMP-OVA and PMP-SYK served in these experiments as examples of the PMP-based protein and peptide ligands, respectively (Figure 2C and 2D). We measured their ability to displace the radioactive tracer, PMP-BSA, from the 1-15F, R1325AF, 1-8F, and 1-3F mini-receptors immobilized on M2 α-FLAG resin. Values for IC_50_ and RBAs calculated in these experiments demonstrate that both PMP-OVA (Figure 2C) and PMP-SYK (Figure 2D) bind 1-15F, the mutant receptors, and the deletion constructs with similar affinities. Thus, these data suggest that the high-affinity interaction of the PMP-ligands must occur through a bivalent M6P-based mechanism via intermolecular cross-bridging of domain 3 M6P-binding sites on two or more M6P/IGF2R monomers.

**Figure 3:**
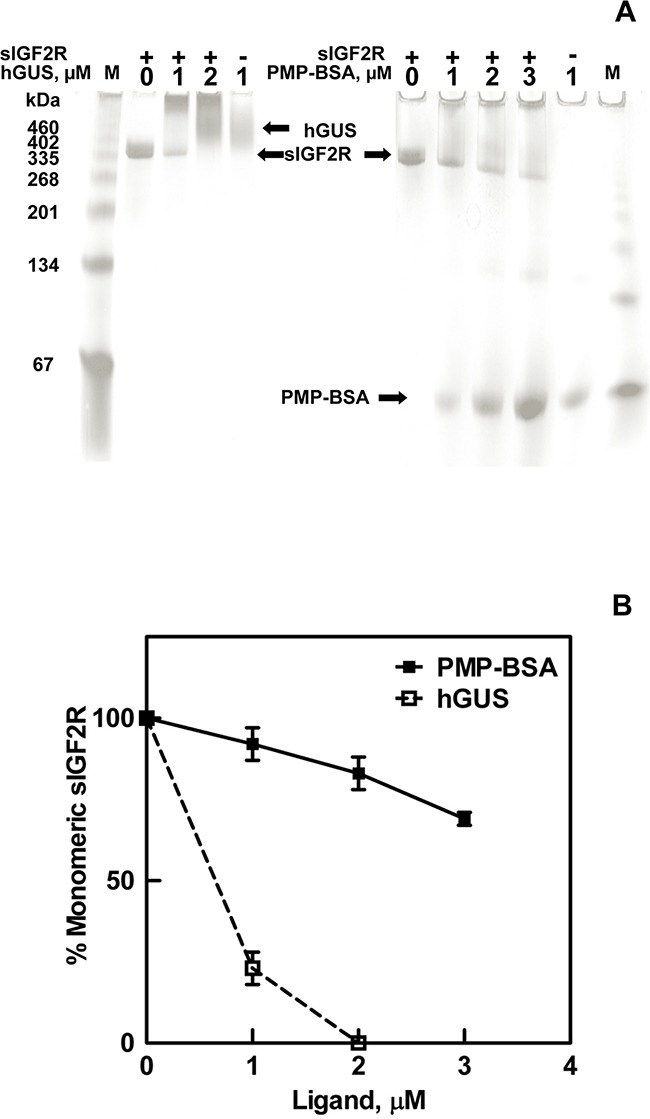
Ligand-induced oligomerization of the M6P/IGF2R Soluble M6P/IGF2R (sIGF2R, 0.4 μM) was incubated at room temperature for 90 min with the indicated concentrations of hGUS or PMP-BSA. A) Samples were run on 4-20% gradient gels, pH 6.8 under native conditions (absence of sodium dodecyl sulfate). BSA run as a standard migrated as a monomer as well as a series of oligomers. Gels were stained with Commassie blue and imaged. B) Densitometric analysis of the sIGF2R monomer bands that remain after incubation with the indicated concentrations of hGUS or PMP-BSA are represented in the line graph.

Further evidence that PMP-ligands bind to the dimeric rather than monomeric forms of M6P/IGF2R was obtained through an assay that permitted visualization of the receptor. Thus, we developed a native gel-shift assay using purified soluble bovine M6P/IGF2R (sIGF2R) incubated in the presence or absence of purified hGUS, as a positive control, or PMP-BSA (Figure 3A). These data demonstrate depletion of the monomeric receptor band as a function of increased multivalent ligand concentration. Co-incubations of hGUS and sIGF2R caused a complete shift of the receptor band to a mass that appeared at the top of the gel and was too large to be clearly resolved (Figure 3A and 3B). This was consistent with formation of a trimeric complex, consisting of a 2:1 mole ratio of M6P/IGF2R to hGUS, which would have an estimated mass of ~ 900 kDa. In this assay, the critical concentration for hGUS-induced oligomerization of all M6P/IGF2Rs appears to be between 1-2 μM, as 1 μM hGUS induced dimerization of 77% of the receptor with no apparent unbound hGUS. Doubling the hGUS concentration to 2 μM completely depleted the band corresponding to ligand-free monomeric sIGF2R, while a band corresponding to free hGUS appears at a slightly higher molecular mass. PMP-BSA also shifted the receptor band to a higher apparent mass; however, even at its highest concentration, PMP-BSA only depleted 31% of the receptor band (Figure 3B). This result is consistent with reports that PMP-BSA binds the M6P/IGF2R with a lower affinity than hGUS [35, 36]. Because a 1:1 complex between PMP-BSA and receptor would have a mass of 400 kDa (readily detectable on these gels), these experiments suggest that PMP-BSA must also have been bound to at least a dimer of M6P/IGF2R, as it was not observed in the separating gel. However, unlike hGUS, PMP-BSA did not completely shift the monomeric sIGF2R to a higher-order oligomeric structure. This may relate to the α(1,2) linkage between the ultimate and penultimate M6P units on the oligosaccharides of the natural, preferred ligand, hGUS, whereas the PMP-ligands give up some affinity due to the α(1,3) linkage [37]. Thus, although PMP-ligands bind with high affinity and cross-bridge the M6P/IGF2R, the orientation of the M6P is not preferred, which may account for differences in stabilization of the dimeric M6P/IGF2R. Furthermore, hGUS may be able to establish other contact points that aid in initiation of the dimer in a cell-free assay whereas the PMP-ligands may be relying solely on M6P-mediated interactions with the receptor. These data suggest that both hGUS and PMP-BSA can cross-bridge the M6P/IGF2R to induce the formation of or stabilize pre-formed oligomeric structures of very high molecular mass.

In summary, the binding characteristics of the panel of PMP-based ligands are consistent with bivalent interaction with the receptor, and the evidence favors a stoichiometry of 1 bivalent ligand to 2 M6P/IGF2R partners, with binding mediated by the repeat-3 M6P-binding domains of each monomer. We conclude that the three-dimensional structures of the ligands' protein scaffolds have the geometry to permit simultaneous interaction of the functional end-groups of the PMP chains with two repeat-3 sites of the dimeric M6P/IGF2R. To complete the proof-of-principle assessment of these ligands, we then tested whether the interaction was sufficient to mimic that of the natural ligand hGUS in stimulating internalization of IGF-II mediated by the M6P/IGF2R and to inhibit IGF effects in living cells.

### PMP-based ligands inhibit growth of mouse fibroblasts

**Figure 4:**
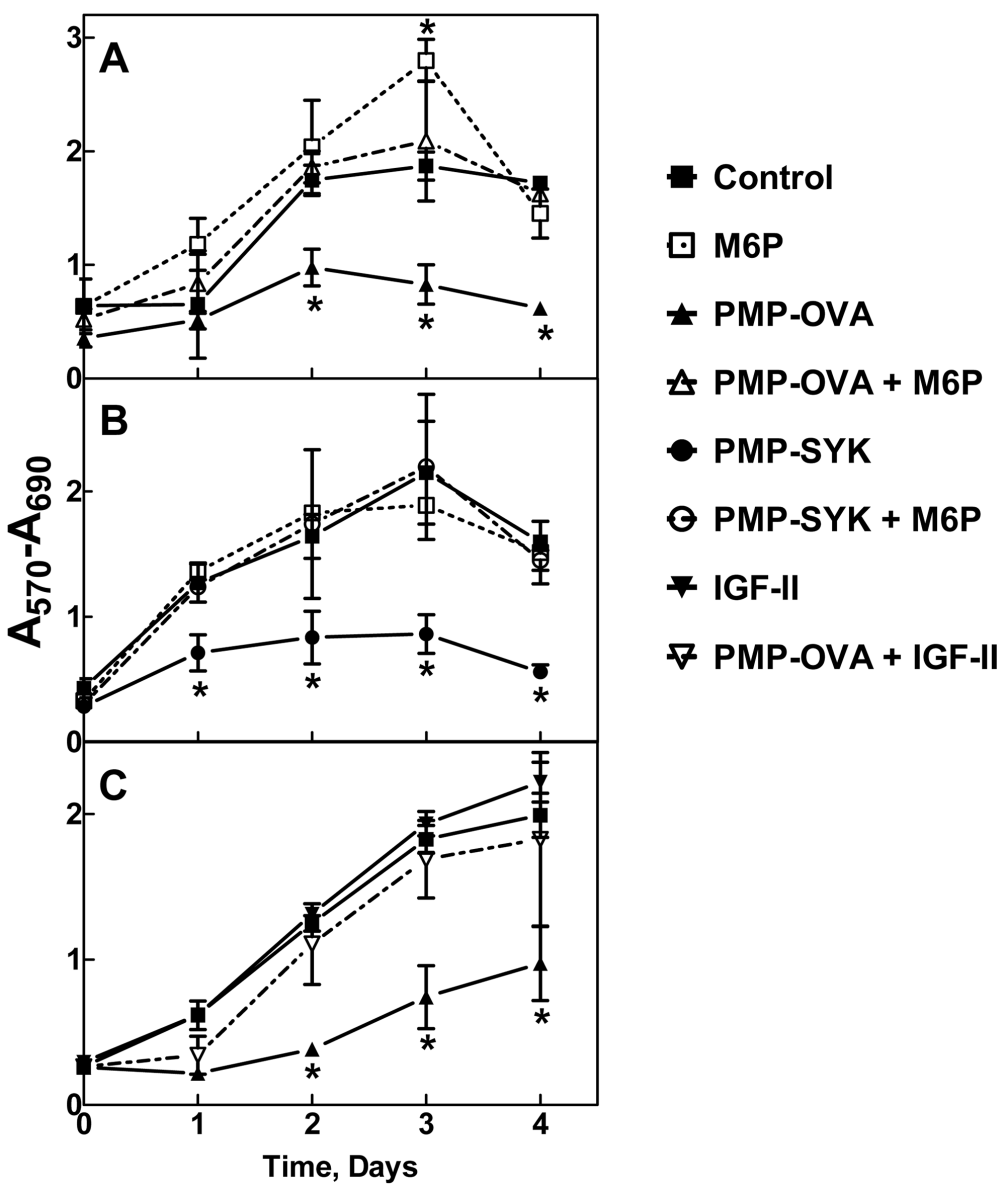
Time course for effects of PMP-OVA or PMP-SYK on L cell growth and survival Mouse L cells overexpressing the human M6P/IGF2R were seeded onto 12-well dishes at 7,500 cells/well, in full serum-containing medium (DMEM, 10% FBS), and were incubated for 24 h before switching to reduced (1%) serum. Cells were then incubated for 24 h under reduced serum conditions for acclimation. At this point, designated day 0, a set of wells was subjected to the MTT assay. In the remaining wells, the various treatments were initiated by adding fresh reduced-serum medium supplemented with M6P (10 mM), M6P (10 mM) + PMP-ligand (10 nM), PMP-ligand (10 nM), IGF-II (10 nM), IGF-II (10 nM) +PMP-ligand (10 nM) or a vehicle control. Incubation was continued for 1-4 days followed by the MTT assay. Wells containing purple formazan were solubilized by adding 500 μL/well of isopropanol; 100 μL from each well was transferred to a 96-well plate and the absorbance values were measured. **A.** 10 nM PMP-OVA ± M6P. **B.** 10 nM PMP-SYK ± M6P. **C.** 10 nM PMP-OVA ± IGF-II. The *ordinate* indicates the absorbance due to reduced MTT at 570 nm corrected for background absorbance at 690 nm plotted as a function of time. Data represent mean ± SEM (n=3); *, *P*<0.05.

To address whether high-affinity binding to the M6P/IGF2R by these small ligands can induce cytotoxic effects *in vitro* similar to those observed with CREG, we utilized a short-term growth assay with mouse L cell fibroblasts stably over-expressing wild-type bovine M6P/IGF2R. This cell line was used by York *et al.* to demonstrate that multivalent M6P ligands induce rapid uptake of IGF-II by the M6P/IGF2R [28]. Cells were incubated with PMP-OVA or PMP-SYK followed by MTT analysis to monitor cell viability over a 4-day time course (Figure 4A and 4B, respectively).

Both ligands produced a notable decrease in cell viability as measured by the decrease in MTT absorbance that was detectable after one day and became more pronounced and statistically significant over the length of the experiment. The monovalent ligand, M6P, produced no effect on its own, but at a concentration of 10 mM, was able to completely abrogate the effect of the multivalent PMP-ligands; this phenomenon is most likely due to its ability to competitively displace the M6P-based ligands from the cell-surface M6P/IGF2R. These data suggest that the decrease in cell growth is attributable to a M6P-dependent binding mechanism with the M6P/IGF2R. Furthermore, our hypothesis predicts these effects are due to increased IGF-II internalization and degradation mediated by the M6P/IGF2R, depriving the cell of IGF-II needed to sustain growth. To address this, a 10 nM concentration of recombinant IGF-II was added back to the cell culture medium, which completely rescued the inhibition on cell viability caused by 10 nM PMP-OVA (Figure 4C). Taken together, these data support our hypothesis by suggesting that the PMP-based ligand-induced decrease in cell viability is dependent on IGF-II. In the time course studies, incubating the cells with IGF-II alone did not stimulate significant increases in cell number beyond that of the untreated control, suggesting at least a modest basal level of IGF axis activation in these cells.

**Figure 5:**
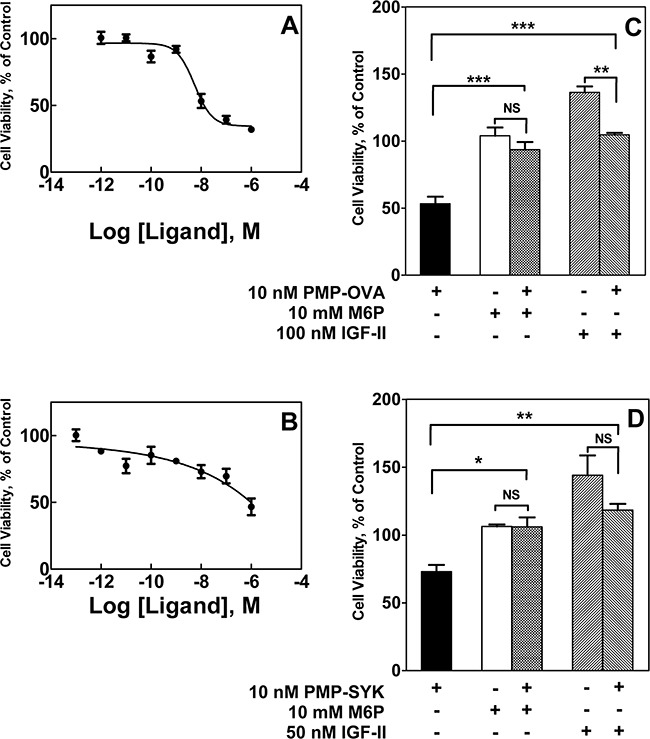
Effect of various M6P/IGF2R ligands on survival of L Cells Mouse L cells were seeded onto 12-well dishes at 3,000 cells/well, in full serum-containing medium (DMEM, 10% FBS), and were incubated for 24 h before switching to reduced (1%) serum. Cells were then incubated for 24 h under reduced serum conditions for acclimation. At this point, designated day 0, a set of wells was subjected to the MTT assay. In the remaining wells, the various treatments were initiated by adding fresh reduced-serum medium supplemented with increasing concentrations of PMP-OVA (1 pM to 1 μM) **A.** or PMP-SYK (100 pM to 1 μM) **B.**, or a vehicle control, with incubation continued for 2 days followed by the MTT assay. Alternatively, L cells were treated with M6P (10 mM), M6P (10 mM) + PMP-ligand (10 nM), PMP-ligand (10 nM), IGF-II (10 nM) or IGF-II (10 nM) +PMP-ligand (10 nM) or a vehicle control with incubation for another 2 days prior to the MTT assay. The effects of PMP-OVA **C.** and PMP-SYK **D.** were normalized to that of the control group to determine the ability to reverse the decreased cell viability caused by the PMP-ligands. The *ordinate* indicates the absorbance due to reduced MTT at 570 nm corrected for background absorbance at 690 nm plotted as a function of time. Data represent mean ± SEM (n=3); *, *P*<0.05; **, *P*<0.01; ***, *P*<0.05.

To test the dose-dependent effects of the PMP-ligands on cell growth, we modified our short-term growth assay by treating the cells with a range of concentrations of PMP-OVA (Figure 5A) or PMP-SYK (Figure 5B) and monitored the response by MTT analysis after 2 days of treatment. A dose-response curve showed that the cells responded to PMP-OVA at concentrations ranging from 1 pM to 1 μM, with the most dramatic effects measured between 10 nM and 1 μM concentrations (Figure 5A), where growth was suppressed by approximately 70%. The mouse L cells exposed to 1 pM to 1 μM PMP-SYK (Figure 5B) also showed a dose-dependent effect on viability, although not as pronounced (growth suppressed by only about 50% at the highest concentration) as the PMP-OVA-treated cells over the two-day time course. Interestingly, treating the cells with 10 nM hGUS decreased cell growth by ~50%, which was comparable to treating the cells with 100 nM PMP-OVA in this experiment (data not shown). The growth-suppressive effect induced by the PMP-ligands was again blocked by saturating concentrations of M6P (Figure 5C and 5D). M6P had no effect on its own, as observed previously (Figure 4). Addition of IGF-II stimulated cell growth and was able to rescue the growth-inhibition effect induced by the PMP-ligands (Figure 5C and 5D). Earlier work by Bock's group produced a synthetic M6P-based tripeptide, which was measured to be a high-affinity bivalent ligand for the M6P/IGF2R [38], but using radioligand-based internalization assays, York *et al.* concluded that this tri-peptide was unable to enhance the rate of M6P/IGF2R endocytosis [28]. Additionally, structure-function analysis by Bock's group revealed that this compound's high affinity for the M6P/IGF2R was not attributed to a M6P-based binding mechanism but through hydrophobic interactions between an anthranoyl group, present on the compounds' ε-amino group of lysine, and a hydrophobic region presumably near the M6P-binding pocket of the receptor [38]. Thus to our knowledge, we have designed the smallest synthetic M6P-based ligand for the M6P/IGF2R that achieves an affinity indicative of bivalent binding that can reduce cell viability in a M6P-based and IGF-II-dependent manner. Our data suggest that the PMP-ligands are binding to the M6P/IGF2R in a M6P-dependent manner, which appears to cause a decrease of IGF-II from the extracellular space by binding to the receptor as a passenger ligand.

### PMP-based ligands inhibit growth of cancer cells *in vitro*

**Figure 6:**
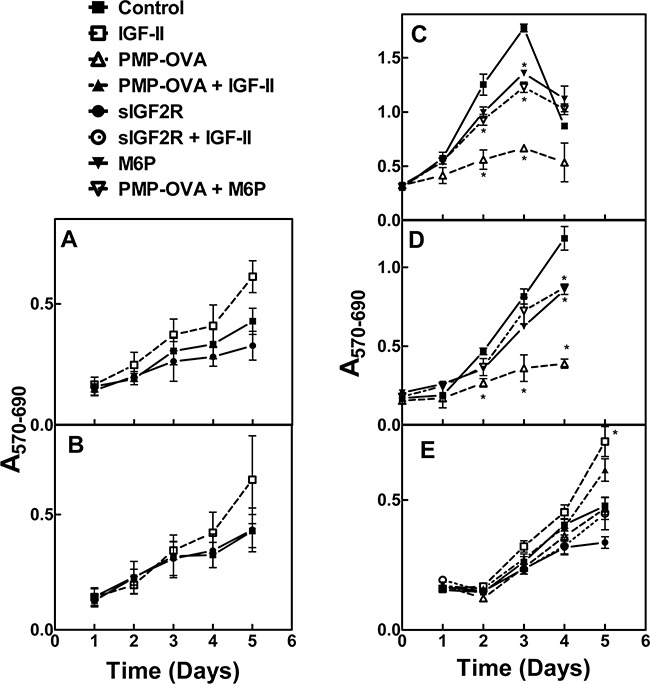
Effect of M6P/IGF2R ligands on cancer cell growth and survival Capan-1 **A.** and S2-013 **B.** were screened for autocrine/paracrine IGF-II loops. Cells were seeded into 96-well plates in complete medium and allowed to attach for 24 h before switching to reduced-serum medium for an additional 24 h for acclimation. Cells were then incubated with 10 nM IGF-II, 10 nM soluble bovine M6P/IGF2R (sIGF2R), or vehicle control and incubation continued for 1-5 days before the MTT assay. JEG-3 **C.** and HuH-7 **D.** cells were seeded in 12-well dishes at 10,000 cells/well, and Capan-1 **E.** cells were seeded in 96-well dishes at 500 cells/well in complete medium and allowed to attach for 24 h before switching to reduced-serum medium. Cells were subjected to treatment with IGF-II (10 nM), PMP-OVA (50 to 200 nM), sIGF2R (10 nM), M6P (10 mM) combinations of these, or vehicle control prior to the MTT assay. The *ordinate* indicates the absorbance due to reduced MTT at 570 nm corrected for background absorbance at 690 nm plotted as a function of time. Data represent mean ± SEM (n=3); *, *P*<0.05.

We next tested whether these compounds can inhibit cell growth in more biologically relevant model systems. We analyzed a series of human cancer cell lines to see if they express the M6P/IGF2R and IGF-II, to determine if these cell lines may have autocrine or paracrine IGF-II loops (Supplementary Figure S1). All the tested cell lines express the M6P/IGF2R, but only a few expressed IGF-II. However, even in the absence of IGF-II expression, these cell lines may be responsive to IGF-II in a paracrine mode rather than via an autocrine loop. Thus, we incubated the pancreatic carcinoma cell lines Capan-1 and S2-013 with 10 nM IGF-II or 10 nM soluble M6P/IGF2R (sIGF2R) for 1 to 5 days to determine if they are IGF-II-responsive and if they are IGF-II growth-dependent, respectively, and measured their viability by MTT analysis (Figure 6A and 6B). Both cell lines responded to exogenous IGF-II in terms of an increase in viable cell number, but only Capan-1 showed decreased cell viability when incubated with the ligand trap sIGF2R. This suggested that Capan-1 exhibited IGF-II dependency that could be inhibited by decreasing the bioavailability of IGF-II in the cell milieu. We also incubated HuH-7 human hepatocellular carcinoma cells, JEG-3 human choriocarcinoma cells, and Capan-1 cells with PMP-OVA and analyzed effects on viability by MTT analysis (Figure 6C-6E). Several studies have demonstrated that HuH-7 and JEG-3 cell lines express M6P/IGF2R along with all other components of the IGF axis, and they exhibit an IGF-II-driven autocrine loop [39–41]. In our laboratory, immunoblot and RT-PCR analysis of M6P/IGF2R and IGF-II expression, respectively, revealed that HuH-7 cells express the M6P/IGF2R and IGF-II, while SK-N-AS cells express the M6P/IGF2R (Supplementary Figure S1). The human neuroblastoma cell line SK-N-AS also expresses IGF-II (data not shown); thus, these cells are also suitable models for our experiments. Initially, HuH-7 and JEG-3 cells were treated with 10 nM PMP-OVA, but we did not observe substantial decreases in the number of viable cells. Further investigation revealed that M6P/IGF2R expression levels in these cells, as detected by immunoblot analysis, were much lower than that of the mouse L cells engineered to over-express the receptor. To compensate for the decreased plasma membrane receptor density and potentially lower M6P/IGF2R internalization capacity in these cells, we increased the dose of PMP-OVA to a level exceeding the IC_50_. Thus, treatment of cells with 200 nM PMP-OVA did produce a decrease in cell growth similar to the effect observed with 10 nM PMP-OVA on L cells (Figure 6C and 6D). Capan-1 cells were treated with 50 nM PMP-OVA to assess the growth suppressive-effect over a five-day period. PMP-OVA decreased cell growth but not as dramatically as with the mouse L cells (Figure 6E). The addition of M6P (10 mM) rescued the growth-suppressive effect, indicating that PMP-OVA was functioning in an M6P-dependent manner. Capan-1 cells showed decreased cell viability in response to incubation with 50 nM PMP-OVA, although not quite to the extent as 10 nM sIGF2R (Figure 6E). However, adding exogenous recombinant IGF-II (10 nM) rescued the growth-suppressive effect when either PMP-OVA or sIGF2R was used (Figure 6E). These data showed that the effects of the PMP-based ligands can be observed in three IGF-II-responsive cancer cell lines, suggesting the decrease in cell growth is not an artifact of high M6P/IGF2R expression in L cells. We have also observed such effects in several other cancer cell lines, including SK-N-AS (below).

In contrast to experiments with sIGF2R in detergent solution (Figure 3), in which PMP-BSA struggled to stabilize the sIGF2R in a dimer, cell-based experiments suggest that even the smallest PMP-based ligand, PMP-SYK, is capable of producing a biological effect, decreasing cell viability by ~70% in mouse fibroblasts, HuH-7 and JEG-3 cell models. Interestingly, we found that, in general, the PMP-based ligands are not as efficient as hGUS at promoting dimerization of the M6P/IGF2R (Figure 3), although they are just as effective at decreasing cell viability (Figure 6). A M6P-phosphodiester binding site was identified in domain 5 of the receptor and exhibits a 60-fold increase in M6P-phosphodiester binding affinity for the receptor over M6P-phosphomonoester binding [11]. A “high-uptake” form of hGUS purified from spleen that differed in charge and M6P content from other sources of hGUS was identified, and the M6P content correlated with the rate of internalization by fibroblasts, where 63% of the “high-uptake” hGUS had one phosphate in a diester linkage to α-linked *N*-acetylglucosamine, most likely binding to domain 5 [12, 42]. Taking these data together with work done by Bohnsack *et al.* and Song *et al.*, phosphodiester-based binding to the receptor, although not as high-affinity as monoester binding, may be important for stimulating rapid internalization of the receptor [14, 43]. Collectively, these interesting observations suggest that M6P-based ligand-induced rapid internalization is an important function of the M6P/IGF2R that merits further study.

### PMP-based ligand effects on apoptosis and mitogenic signaling

**Figure 7:**
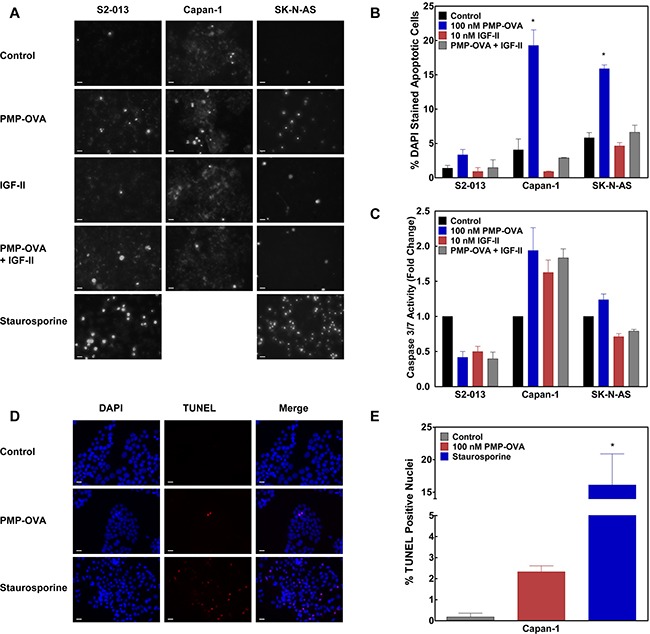
Effects of PMP-OVA and IGF-II on apoptosis responses in cancer cell lines Panel A: DAPI analysis of apoptotic nuclei. S2-013, Capan-1, and SK-N-AS cells were seeded into 24-well plates in complete medium and allowed to attach for 24 h before switching to reduced-serum medium containing the various treatments. Cells were treated with PMP-OVA (200 nM), IGF-II (10 nM), or vehicle control for 24 h before DAPI (5 μg/mL) was added directly to the medium and incubation continued for 20 min. Representative fields were captured under fluorescent and phase-contrast channels using a fluorescent microscope. The number of apoptotic nuclei (evidenced by fragmentation) and total cells (counted under phase-contrast illumination) were counted manually from a field of 70-150 total cells. The *bar* indicates 20 μm. Data for quantification **B.** of DAPI staining from the three cell lines have been plotted, (n=3). Values for PMP-OVA treatment in Capan-1 and SK-N-AS cells were significantly different compared to control, *P* <0.001. The quantification of caspase 3/7 activation **C.** was used as a biochemical marker for apoptosis. S2-013, Capan-1, and SK-N-AS cells were seeded into 96-well, black clear bottom plates. Apo-ONE® Homogeneous Caspase 3/7 Activity Assay (Promega) was used per manufacturer's instructions. TUNEL assay **D.** was used as a second measure of apoptosis. Capan-1 cells were seeded into Nunc^TM^ Lab Tek^TM^ II 8-well chamber slides in complete medium for 24 h. Cells were treated with PMP-OVA (100 nM), staurosporine (21 μg), or vehicle control in reduced-serum medium for 24 h. Representative fields were captured under fluorescent channels using a fluorescence microscope. TUNEL-positive nuclei (indicated by a bright red dot) and total cells (DAPI stained) were counted in a field of 100-150 cells. The *bar* indicates 20 μm. Data for quantification **E.** of TUNEL staining from the three cell lines have been plotted. Data represent mean ± SEM (n=3); *, *P*<0.05.

The growth-suppressive effects of PMP-OVA and PMP-SYK on mouse L cells could arise either by inhibition of cell proliferation or by stimulation of a cell death response due to growth factor deprivation, or a combination of both effects. Upon activation of the IGF1R or hybrid of IGF1R/IR-A via IGF-II binding, several different downstream signaling pathways can become activated. One such final end-state effect mediated through the PI3K/Akt survival pathway is decreased apoptosis, or programmed cell death. To determine if PMP-ligands stimulate apoptosis due to IGF-II depletion, we performed a series of experiments to examine apoptosis in cells. Capan-1 and S2-013 pancreatic cancer cell lines and SK-N-AS neuroblastoma cells were seeded into 24-well plates and treated with 100 nM PMP-OVA, 10 nM IGF-II, PMP-OVA + IGF-II, 21 μg staurosporine, or vehicle control (Figure 7). To assess nuclear fragmentation as a measure of apoptosis, we stained the cells with 5 μg/mL DAPI, a fluorescent stain that binds A-T-rich regions of DNA, and tends to accumulate to a higher concentration indicated by increased fluorescence intensity, in cells undergoing apoptosis [44, 45]. The data in Figure 7A are representative images taken under fluorescent microscopy and converted to grayscale. Apoptotic and total cells were counted manually with 75-150 total cells per field. Capan-1 cells treated with 100 nM PMP-OVA exhibited about a 5-fold higher number of apoptotic nuclei compared to the control (Figure 7A and 7B). The addition of 10 nM IGF-II overcame the increase in apoptosis, which returned to the level of the vehicle control, while cells treated with IGF-II on its own had fewer apoptotic nuclei than the negative control. S2-013 cells showed an overall similar response as the Capan-1 cells, with a 2.5-fold increase in apoptotic nuclei over the control with PMP-OVA treatment and a slight but non-significant decrease in IGF-II-treated cells (Figure 7A and 7B). The SK-N-AS cells had a higher resting number of apoptotic nuclei per 100 cells than the other cell lines, yet PMP-OVA increased the number of apoptotic nuclei approximately 3-fold compared to the control. The IGF-II-treated cells had a non-significant reduction in apoptotic nuclei while the add-back of IGF-II again rescued PMP-OVA's pro-apoptotic response (Figure 7A and 7B). The positive control staurosporine increased the number of nuclei in the S2-013 and SK-N-AS cells, but was not used in Capan-1 cells. The ability of M6P to rescue this effect of the PMP-ligands was not tested with this design.

We tried to confirm that apoptosis is induced by PMP-ligand treatment of these cells looking at biochemical indicators of the intrinsic and extrinsic apoptosis pathways. Thus, to assay caspase activation, S2-013, Capan-1 and SK-N-AS cells were seeded into black, clear-bottom 96-well plates. After a 24-hour incubation with 100 nM PMP-OVA, 10 nM IGF-II, PMP-OVA + IGF-II, 21 μg staurosporine, or vehicle control, caspase 3/7 activity was measured according to the manufacturer's instructions. Interestingly, there was no significant increase in caspase activation in the S2-013 cell line, in contradiction to the slight increase in DAPI staining, as all treatment conditions showed lower caspase activity than that of the control (Figure 7C). However, in the Capan-1 cells, all treatment groups (PMP-OVA, IGF-II, and PMP-OVA + IGF-II) showed a small, 1.7- to 2-fold increase in caspase 3/7 activity compared to the negative control. Moreover, SK-N-AS cells showed no apparent change in the caspase 3/7 activity among the various treatment groups (Figure 7C). As a positive control for the apoptosis response, staurosporine treatment caused a 1.5- and 8.5-fold increase in caspase activity in S2-013 and SK-N-AS cells, respectively. From these data, the slight increase in apoptotic nuclei detected in the DAPI staining assay does not appear to be correlated with caspase 3/7 activation, with the possible exception of the Capan-1 cells. The TUNEL assay was used as an additional method to detect apoptosis (Figure 7D and 7E). Capan-1 cells were seeded into Nunc^TM^ Lab Tek^TM^ II 8-well chamber slides in complete medium for 24 h. Cells were treated with PMP-OVA (100 nM), staurosporine (21 μg), or vehicle control in reduced-serum medium for 24 h. The data in Figure 7D are representative images taken under fluorescence microscopy in a field of 100-150 total cells. PMP-OVA treatment increased TUNEL-positive staining from <0.2% of total cells counted to 2.3%, while staurosporine increased TUNEL-positive cells up to 16% of the total. Although the increase in apoptotic cells in the PMP-OVA treatment seems small, the cell-death effect represents a 13-fold increase in TUNEL-positive compared to the control. Due to the pronounced, ~90- fold increase in TUNEL-positive cells by staurosporine, we were unable to show that the difference between the PMP-OVA treatment and control was statistically significant using ANOVA. Furthermore, PARP cleavage as an additional marker for apoptosis showed a 2-fold elevation between the PMP-OVA and negative control in S2-013 and SK-N-AS cells. To put this effect in perspective, the apoptosis positive control (staurosporine) caused a 25- to 30-fold increase in PARP cleavage in S2-013 and SK-N-AS cell lines, respectively. The addition of a protease inhibitor (leupeptin) increased PARP cleavage 5-fold over the control, and co-incubation with leupeptin plus PMP-OVA slightly increased this cleavage (data not shown). Similarly weak or minimal effects of the PMP-based ligands on PARP cleavage were observed in several cell lines (data not shown).

To determine if the PMP-based ligands may influence cell viability by inhibiting IGF-dependent mitogenic and survival signaling, the activation/phosphorylation status of several key effectors in the Akt and ERK pathways were measured by immunoblotting (Supplementary Figure S2). For these experiments, SK-N-AS cells were grown to 70-80% confluence and treated with 100 nM PMP-OVA, 10 nM IGF-II, PMP-OVA + IGF-II, 10 nM sIGF2R, sIGF2R + IGF-II, or vehicle control (HBS) for 24 hours. Whole-cell extracts were then subjected to SDS-PAGE followed by immunoblot analysis. The phosphorylation of Akt at Thr308 or Ser473 was not noticeably different among the treatment groups compared to control (Supplementary Figure S2). However, there was a small increase in ERK1/2 phosphorylation as a result of IGF-II treatment. Nevertheless, PMP-OVA did not affect basal ERK1/2 phosphorylation on its own, and it was unable to counteract this slight effect of IGF-II. Overall, our experiments with SK-N-AS cells and with the other cancer cell lines (data not shown) did not support the hypothesis that the PMP-based ligands inhibited cell proliferation or survival signaling to a great extent under these conditions. At this point, having tested the biological effects of these M6P-based ligands for the M6P/IGF2R in several cell lines having different levels of dependence on the IGF axis for cell growth and survival, it seems that the degree of efficacy differs among cell types. This led us to examine the more fundamental question that was the premise underlying the proposed action of these PMP-based ligands to impact IGF signaling—do these ligands stimulate the M6P/IGF2R to internalize IGF-II and promote its degradation by cells?

### PMP-based ligands deplete IGF-II from the conditioned medium

To determine whether the PMP-ligands promote depletion of the bioavailable IGF-II in the medium bathing cultured cells, we tried various methods until we were able to achieve the most sensitive assay to detect the low concentrations of IGF-II in conditioned medium (CM). Traditional immunoblot analysis of the CM after SDS-PAGE separation was unsuccessful, as the antibodies we used did not have high enough sensitivity. Concentrating the CM by lyophilization and re-dissolution or by centrifugal ultrafiltration did not improve detection by that approach. Previously, our laboratory was able to detect IGF-II in CM using size-exclusion chromatography under acidic conditions coupled with an ^125^I-IGF-II radioimmunoassay [46]. We adapted these methods, concentrating 4-mL aliquots of CM 20-fold and running the acidified soluble protein mixture on a Bio-Gel P10 column. The fractions were then assayed for IGF-II by radioimmunoassay or a radioreceptor assay. Even though these approaches worked well with IGF-II standards, we were unable to achieve sufficiently high sensitivity to detect the small amounts of IGF-II present in the CM with reproducibility (data not shown).

Despite not having yet found an assay sensitive enough to detect minute differences in IGF-II in the CM of cultured cells, we decided to take a direct approach to assay degradation that is also an indirect measure of internalization. Thus, we developed an ^125^I-IGF-II-based internalization/degradation assay that takes advantage of the inherent acid-insolubility of intact IGF-II polypeptide relative to the cellular products of IGF-II degradation, which are expected to be soluble in concentrated acid. For the assay, we added 0.3 nM ^125^I-IGF-II tracer to low-serum (3%) growth medium at time zero of the treatment period, and then tracked recovery of intact ^125^I-IGF-II for up to 48 h by measuring the trichloroacetic acid (TCA)-soluble and -insoluble radioactive material remaining in the CM over time. Intact IGF-II precipitates with the carrier proteins (mainly albumin) when the CM is treated with 46% TCA while any low-molecular-weight (degraded) radiolabeled fragments of IGF-II are recovered in the supernatant fraction. The rationale behind this approach is that M6P/IGF2R-mediated uptake of the labeled IGF-II by cells followed by its intracellular degradation would tend to deplete the intact IGF-II from CM and proportionally increase the amount of radiolabeled breakdown products released back into the CM. Additionally, we added 1 nM unlabeled IGF-II to the cells in an attempt to protect the radioligand from adsorption to matrix surfaces that bind IGF-II, such as vitronectin within the EC matrix [47] or IGFBPs in the medium, and to simulate a reasonable concentration of pericellular IGF-II as might be encountered *in vivo*.

Testing our approach in a cell-free system demonstrated that the ^125^I-IGF-II is precipitable in TCA and is stable throughout the course of the study, with 19% of the input counts in the soluble fraction and 70% in the insoluble fraction at 0 h. Over the course of a 4-day incubation at 37°C, the insoluble counts shifted to the soluble form at a rate of 1% per day. Thus, changes in TCA solubility exceeding that rate were considered to be a direct result of the actions of the cells. As another control, we included an IGF-II treatment group for these studies, in which a 100-200 nM concentration was chosen in order to inhibit degradation of ^125^I-IGF-II by out-competing the radioligand for the available receptor binding domains; thus, there should be more radioactive counts in the CM corresponding to intact ^125^I-IGF-II as the assay progresses. Other negative controls in these assays were the inclusion of sIGF2R as a ligand trap or of M6P to inhibit M6P/IGF2R-mediated binding of PMP-OVA.

**Figure 8:**
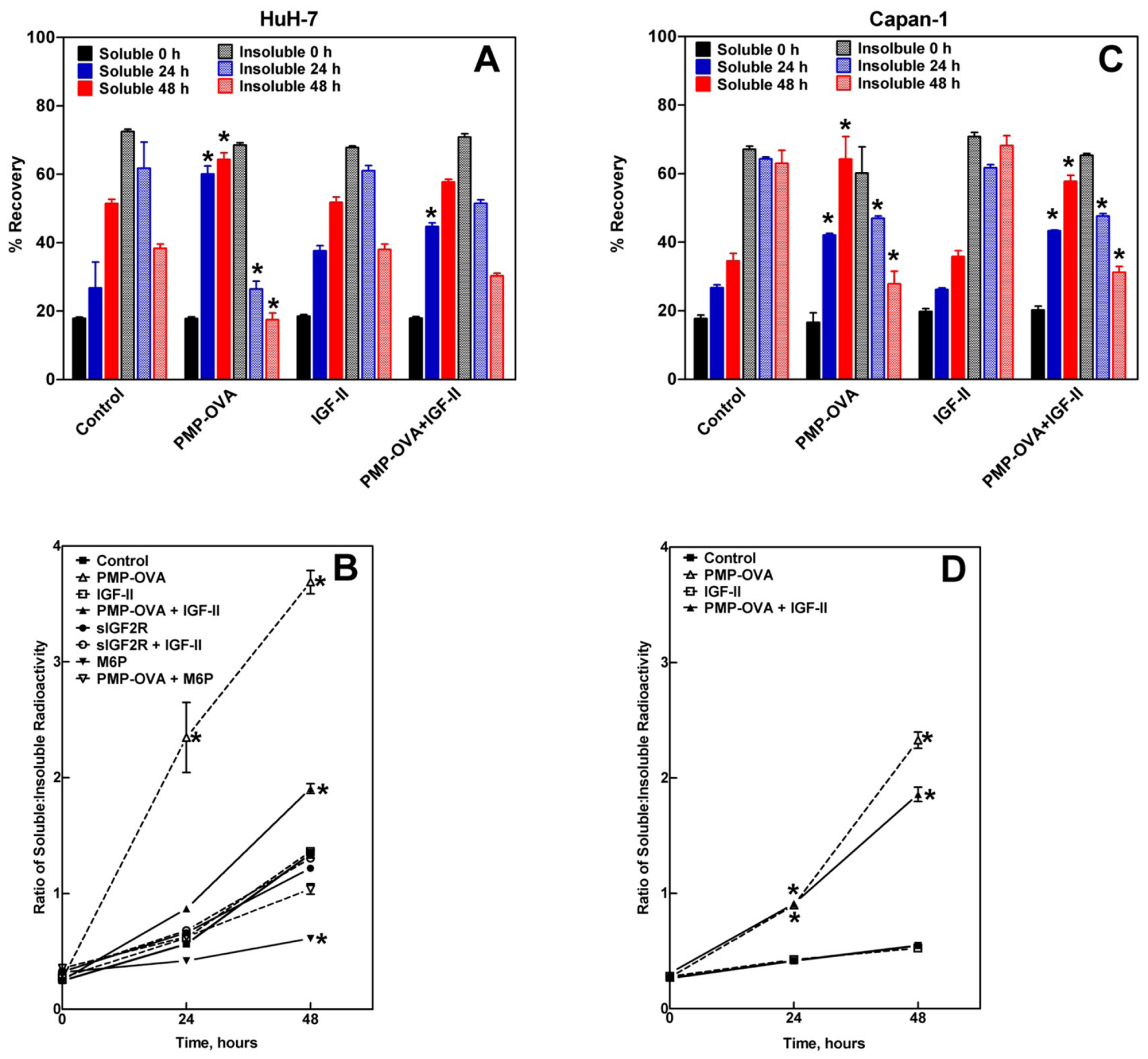
Precipitation of ^125^I-IGF-II from conditioned medium by trichloroacetic acid (TCA) HuH-7 and Capan-1 cells were seeded into 24-well plates in complete medium and allowed to attach for 24 h before switching to reduced-serum medium supplemented with the various treatments plus 0.3 nM ^125^I-IGF-II and 1 nM cold carrier IGF-II. The treatments were: IGF-II (100 nM), PMP-OVA (100 nM) ± IGF-II (100 nM), sIGF2R (10 nM) ± IGF-II (100 nM), M6P (10 mM) ± PMP-OVA (100 nM), or vehicle control. At the indicated times, aliquots of the conditioned medium were treated with TCA (46% final concentration). After incubation on ice for 1 h, the samples were centrifuged and the amounts of radioactivity recovered in the pellet and supernatant fractions were measured. These data were converted to percent recovery based on input radioactive IGF-II and plotted as indicated in the HuH-7 **A.** and Capan-1 **C.** cells. PMP-OVA treatment caused a significant increase of radioactive material in the soluble fraction and a significant decrease in that of the insoluble fraction at both 24 h and 48 h compared to the control at 24 h and 48 h. Data represent mean ± SEM (n=4); *, *P*<0.001. The ratio of the TCA-soluble to TCA-insoluble radioactive material recovered at each time point was calculated and plotted vs. time for each treatment group in HuH-7 **B.** and Capan-1 **D.** cells. Data represent mean ± SEM (n=3); *, *P*<0.01.

Our results show that using this assay, we were able to indirectly detect internalization of ^125^I-IGF-II and directly detect degradation of the radiolabeled growth factor by analysis of CM; we obtained data from several cell lines, but our data set is most complete for HuH-7 (Figure 8A and 8B) and Capan-1 (Figure 8C and 8D) cells. The data show that ^125^I-IGF-II was internalized, degraded, and secreted back into the medium progressively over the course of the assay, as seen by a time-dependent increase in radioactive counts in the TCA-soluble fraction and a corresponding decrease of counts in the TCA pellet (Figure 8A and 8C). With the addition of PMP-OVA, there was an increase in IGF-II internalization as indicated by a sharp drop in the amount of TCA-precipitable IGF-II compared to the control after 1-2 days of incubation with the PMP-OVA. Additionally, there was a more pronounced increase of radioactive material in the TCA supernatant (degraded IGF-II) when PMP-OVA was added to the cells. Interestingly, the high concentration of IGF-II (100-200 nM) behaved similarly to the control instead of protecting the ^125^I-IGF-II from degradation. However, inclusion of IGF-II with PMP-OVA decreased ^125^I-IGF-II internalization/degradation, suggesting a protective effect of the unlabeled growth factor (Figure 8B and 8D). Furthermore, M6P co-incubation with PMP-OVA partially rescued the effect on ^125^I-IGF-II internalization/degradation, and sIGF2R inhibited the internalization of the ^125^I-IGF-II (Figure 8B). In all treatment groups throughout the course of the assay, the radioactive material in the TCA supernatant and pellet yielded approximately an 80-100% overall recovery in amount of total radioactivity added to the assay. Therefore, from our ^125^I-IGF-II internalization/degradation assay, we conclude that PMP-ligands are able to bind to the M6P/IGF2R and promote internalization and degradation of extracellular IGF-II as a passenger ligand. Han *et al.* were able to measure internalization of IGF-II with overexpression of CREG in human vascular smooth muscle cells, using a mouse IGF-II ELISA kit [48]. Furthermore, knock-down of CREG or blocking the ability of IGF-II to bind to the M6P/IGF2R using neutralizing antibodies reversed the IGF-II internalization effect. However, although the study of Han *et al.* validated a role for the M6P/IGF2R in CREG-induced reduction in IGF-II in CM of cells, they did not report whether the effects were mediated by a M6P-based mechanism.

The human *IGF2* gene is maternally imprinted, and loss of imprinting or gene amplification are important contributors to many cancers, including those of the ovary, liver, breast, colon, prostate, and pancreas [40, 49–53]. Re-expression arises from reactivation of the downstream promoters by altering their methylation status [51, 54–56). It has been suggested that decreasing IGF-II concentration in the extracellular milieu can suppress IGF-II-induced growth and proliferation in such cancers [39, 57–61]. It is in this context that we have tried to improve understanding of the trafficking and dimerization functions of the M6P/IGF2R to create therapeutic interventions for the treatment of IGF-II-dependent cancers. Several studies have shown that loss of function of the M6P/IGF2R leads to increased cancer risk [20]. *In vivo* studies revealed that mutation or down-regulation of the M6P/IGF2R in mice led to overgrowth and perinatal lethality; however, *M6p/Igf2r* mutants in mice are rescued and viable when a second mutation in *Igf2* or *Igf1r* is present, although these mice are smaller than their wild-type counterparts [57, 62–64). It was also observed that loss of M6P/IGF2R expression results in elevated IGF-II levels in sheep [62]. The importance of the M6P/IGF2R in development was confirmed in *M6p/Igf2r* knockout mice using the Cre/loxP system, in which complete knockout resulted in perinatal lethality while tissue-specific knockout in the liver or skeletal and cardiac muscle produced viable mice [65]. Along with loss-of-function mutations in the receptor, loss of heterozygosity at the *M6P/IGF2R* locus has been implicated in many cancers, such as those of the liver, breast, lung, ovaries, and adrenal cortex [2, 53].

The frequent observation that IGF-II overexpression by tumor cells or within the tumor microenvironment serves to maintain tumor growth and progression has motivated efforts to design anti-tumor strategies to restrict IGF-II. There are at least three approaches to regulate bioavailability of IGF-II at the protein level: neutralizing monoclonal antibodies (mAbs) against IGF-I and –II; high-affinity ligand traps; and multi-dentate M6P-based ligands targeting the M6P/IGF2R. The mAbs function by binding the IGFs and preventing their subsequent binding and activation of the IGF1R, and are able to inhibit without affecting systemic glucose homeostasis [66]. For example, MEDI-573 is the first humanized mAb capable of neutralizing both IGF-I and -II that shows some early promise in clinical trials [67]. One of the challenges of this strategy is that binding of IGF-II by the mAb has single-use efficacy. The mAb-IGF complexes, once formed, are likely adsorbed and degraded, with no built-in mechanism for recycling the mAb for further use.

Another anti-IGF therapeutic approach is to exploit naturally occurring IGF binding molecules to sequester IGF-II, by means of ligand traps. Modified forms of soluble M6P/IGF2R and various IGF binding proteins (IGFBPs) have been designed to function in this manner. Soluble M6P/IGF2R administered to an IGF-II-overexpressing colon cancer mouse model rescued the cancer phenotype [68]. Domain 11 of the M6P/IGF2R encompasses the primary IGF-II-binding domain, and a single substitution of Glu1554 to Lys increases the affinity for IGF-II by 6-fold; fusion with the carboxyl-terminal human IgG1 Fc domain further increases the affinity for IGF-II as well as the half-life of the ligand trap [69]. Treatment with this agent inhibited proliferation of HaCaT human keratinocytes and *Igf2*^−/−^ mouse embryonic fibroblasts. In a recent report, the Hassan group has used further mutagenic alterations of the ligand-interacting surface of M6P/IGF2R domain 11 to generate a refined and improved version of their IGF-II ligand trap, and they have now moved testing of this agent to a xenograft tumor model [70]. Thus, they showed that treatment with this new ligand trap could inhibit tumor formation by a retrovirally transformed SKNMC Ewing sarcoma cell line that had been selected for dependence on an autocrine IGF-II growth loop [70]. Their data support the conclusion that the cytostatic effects of such IGF-II ligand-trapping agents may be efficacious in combined anti-tumor therapeutic strategies. Additionally, a modified, protease-resistant IGFBP-2 has been shown to bind IGF-I and –II with high affinity and inhibit proliferation in breast cancer cells [71]. The difference between using a modified IGFBP over a modified M6P/IGF2R is that the IGFBP can bind and sequester both IGF-I and -II, potentially leading to diabetogenic effects, whereas the receptor-based traps sequester only IGF-II. Nevertheless, ligand traps have the same limitations as mAbs in that they are single-use agents that must be provided in a continuous manner to bind newly secreted IGF-II to maintain the inhibitory state of reduced pericellular IGF-II bioavailability.

Lastly, bi- or multi dentate M6P-based ligands function to decrease the bioavailability of IGF-II by targeting and stabilizing the dimeric structure of M6P/IGF2R. Thus, IGF-II binds to domain 11 of cell-surface M6P/IGF2R and is internalized and trafficked to the lysosome, where the ligand dissociates and undergoes degradation. In terms of therapeutic strategy, M6P-based ligands may have an advantage over the ligand trap approach in that the agent may be recycled along with the receptor while IGF-II is degraded. Additionally, the M6P-based ligands can be designed as small molecules that resist deactivation by phosphatases, through substitution of mannose 6-phosphonate linkages [32, 72], and that resist proteolysis by linking functional groups through a non-protein tether. In the present study, we have tested a set of M6P-based ligands built on protein-based scaffolds to provide proof-of-principle support for this approach. However, refining this approach demands that several challenges and drawbacks be overcome, including demonstration that activating M6P/IGF2R activity does not deplete the receptor, causing down-regulation and decreasing its availability at the cell surface. This type of agent also must be validated for its ability to promote M6P/IGF2R activity even in the presence of M6P-tagged potential ligands expected to exist naturally in the pericellular region. Finally, with the ligand-based approaches, it will be necessary to rule out their potential IGF-independent activity, such as those that might arise from displacement of lysosomal enzymes from their normal cellular itinerary.

Overall, the present study strengthens our understanding of high-affinity M6P-based multivalent ligand binding, which appears to be necessary to promote dimerization and internalization of the M6P/IGF2R, but may not always be sufficient to alter cell death or cell cycle progression universally in cancer cells. Just as important to the cells' response to this type of growth-suppressive approach is the degree to which the cells are dependent on the IGF axis, and especially on IGF-II, for proliferation and survival. Nevertheless, this study shows that the approach to use high-affinity M6P-based ligands to stimulate M6P/IGF2R-based depletion of pericellular IGF-II is technically feasible, and that the M6P/IGF2R may be a useful chemotherapeutic target in this unusual sense. Efficacy may be improved by coupling this approach with other types of chemotherapeutic agents in combination therapy. It would also help to develop a method to quantify dimerization of the receptor *in situ* in the plasma membrane, or at least to demonstrate definitively that it occurs. This is a very important experiment in this field that would support the underpinning mechanism for the effects of ligands that are proposed to stabilize the dimeric structure and influence receptor dynamics.

To date, only a few studies have targeted the M6P/IGF2R for therapeutic purposes. Among them are the use of human serum albumin (HSA) modified with M6P moieties, which have been used to target the M6P/IGF2R as a conduit to deliver both anti-fibrotic and anti-cancer drugs into cells [73–75]. Additionally, several laboratories have successfully developed synthetic M6P-mimetics that exhibit equivalent or slightly better affinity for the M6P/IGF2R than the naturally occurring monovalent ligand, M6P [32, 33, 72, 76]. Moreover, Jeanjean *et al.* demonstrated that these M6P-mimetics have no detectable cytotoxicity on cells [76]. However, to our knowledge there are no synthetic M6P-based ligands capable of binding the M6P/IGF2R to produce bivalency, such as the affinity comparable with a natural ligand like hGUS. Therefore, the goal of this work was to develop a panel of bi- and multi-dentate M6P-based ligands that mimicked the affinities and activities reported for the natural M6P/IGF2R ligands, hGUS and CREG. Based on our data and the available literature, we speculate that dimerization and internalization of the receptor are promoted through multiple contacts made between M6P-based ligands and the M6P/IGF2R's ectodomain. Upon stabilization of the dimeric receptor, the passenger ligand IGF-II can be internalized and degraded, presumably in the lysosome, thereby limiting the bioavailability of this ligand for the mitogenic receptor, IGF1R. We conclude that, to the best of our knowledge, we have designed the first synthetic, high-affinity M6P-based ligands that stimulate M6P/IGF2R-mediated internalization and degradation of IGF-II, suggesting its promise as a novel chemotherapeutic agent.

## MATERIALS AND METHODS

### Materials

Bio-Gel P-10 was obtained from Bio-Rad (Richmond, CA). Recombinant human IGF-II was purchased from Bachem (Torrance, CA). Carrier-free Na^125^I was acquired from PerkinElmer Life Sciences (Waltham, MA). Bovine serum albumin (A-7906), chicken egg white albumin (ovalbumin, OVA, A-5503), insulin (I-6634), D-mannose 6-phosphate (M6P) disodium salt (M-6876), β-D-glucose 6-phosphate (G6P) monosodium salt (G-7879), cyanogen bromide (CNBr)-activated-Sepharose 4B (C-9142), Sephadex G-50 fine, quaternary aminoethyl (QAE)-Sephadex A25 and chloramine-T were obtained from Sigma (St. Louis, MO). Lysyl-tyrosyl-lysine (KYK) acetate salt (#153148) was purchased from MP Biomedicals, Inc. (Solon, OH). The tripeptide Ser-Tyr-Lys (SYK) was from Bachem (H-4985.0250), IODOGEN tubes, Slide-A-Lyzer® dialysis cassettes, and Precise™ Protein Gels were from Pierce (Rockford, IL). Cell culture media and reagents were from Life Technologies (Carlsbad, CA), while fetal bovine serum was from Atlanta Biologicals (Flowery Branch, GA). Pentamannosyl 6-phosphate (PMP) was prepared from the native Y-2448 *O*-phosphomannan of *Pichia holstii* according to the method of Murray and Neville, as modified by Ferro *et al.* [77, 78]. The pCMV5 vector was provided by Dr. David W. Russell (University of Texas Southwestern Medical Center, Dallas, TX). The 8.6-kilobase-pair human M6P/IGF2R cDNA and purified human β-glucuronidase were provided by Dr. William S. Sly (St. Louis University Medical Center, St. Louis, MO). Mouse monoclonal anti-FLAG M2 IgG (#F3165) and mouse monoclonal anti-β-actin (#A5441) and the M2 agarose affinity gel were purchased from Sigma. The mouse monoclonal anti-CD222 IgG MEM-238 (#ab8093) (referred to as α-M6P/IGF2R) was purchased from Abcam (Cambridge, MA). The rabbit monoclonal α-pAkt(Thr308) (#4056), mouse monoclonal α-pAkt(Ser473) (#4051), rabbit polyclonal α-Akt (#9272), rabbit polyclonal α-pERK (#9101) antibodies were from Cell Signaling Technologies (Danvers, MA). Rabbit polyclonal α-ERK (#sc-94) was from Santa Cruz Biotechnology (Santa Cruz, CA). IGF-II antibody (#PAC1) was purchased from Cell Sciences (Canton, MA). Peroxidase AffiniPure goat anti-mouse IgG (light chain) (#115-035-174) secondary antibody was purchased from Jackson Immunoresearch Lab (West Grove, PA). Pierce® goat anti-rabbit (#31460) IgG (H+L) horseradish peroxidase-conjugated secondary antibody and the enhanced chemiluminescence reagent SuperSignal® West Femto Maximum Sensitivity Substrate kit were purchased from Thermo Scientific (Waltham, MA). Apo-ONE® Homogeneous Caspase 3/7 Activity Assay was from Promega, (Madison, WI).

### Preparation of ^125^I-labeled tracers

Iodination of IGF-II was carried out by a modification of the chloramine-T method (to a specific activity of ~30-60 Ci/g) as described by GroPep (Bulletin #3001: Procedure for Iodination of IGFs; http://www.gropep.com.au/index.php/article/view/109/1/21). Briefly, aliquots (6 μg in 15 μL 5 mM HCl) of recombinant human IGF-II were mixed with 60 μL of 0.3 M sodium phosphate buffer, pH 7.4, and combined with 2.0-2.5 mCi (~20 μL) of carrier-free Na^125^I. The resulting reaction mix was combined with 20 μL of a 0.4 mg/mL solution of chloramine-T, mixed thoroughly, and incubated for 60 s at room temperature (RT). At the end of the incubation, the reaction was quenched by addition of a 0.6 mg/mL solution of sodium metabisulfite (20 μL). The sample was thoroughly mixed, allowed to incubate for 5 min at RT, then prepared for size-exclusion chromatography by adding 200 μL column buffer (phosphate-buffered saline (PBS); 10 mM sodium phosphate, pH 7.4; 150 mM NaCl) containing 1% BSA. The ^125^I-IGF-II was separated from the free iodine on a 30-mL Sephadex G-50 column equilibrated with column buffer. The fractions containing radiolabeled IGF-II were collected, pooled, and stored at −20°C. Aliquots of PMP-BSA (25 μg) or hGUS (15 μg) were iodinated to specific activities of ~50-150 Ci/g by incubation in 0.3 M sodium phosphate buffer, pH 7.4, with 2 mCi Na^125^I in IODOGEN tubes pre-coated with the oxidizing agent, 1,3,4,6-tetrachloro-3a,6a-diphenylglycoluril, for 15 min, according to the manufacturer's specifications. The product was separated from free iodine on a Sephadex G-50 column equilibrated with PBS + 1% BSA. The iodinated PMP-BSA and hGUS were collected from the flow-through fractions and stored at −20°C.

### Preparation of PMP-sepharose

To make PMP-Sepharose, PMP was first conjugated to (p)-aminophenyl-ethanolamine (PAPEA), also called 2-(4-aminophenyl)-ethylamine, to provide a primary amine for coupling to cyanogen bromide (CNBr)-activated Sepharose 4B (Sigma) as previously described [79]. PMP, sodium salt (50 mg) was incubated with 0.5 mL of PAPEA for 16 h at RT. To complete the coupling reaction, 12 mg of NaBH_3_ was dissolved in 1.5 mL of 100% ethanol and added to the reaction. This mixture was incubated at RT for 5 h, and remaining NaBH_3_ was quenched on ice with the addition of 4 mL H_2_O and glacial acetic acid to a pH of 5.6. The product was lyophilized, and PMP-PAPEA was separated from unreacted PAPEA on a 30 mL Sephadex G10 column equilibrated in 50 mM ammonium acetate, pH 6.0. The purified PMP-PAPEA was lyophilized and stored −20°C until use. A PMP-Sepharose resin coupled to a density of 10 mg protein per ml resin was prepared using cyanogen bromide-activated Sepharose 4B following the manufacturer's instructions

### Cell culture

Mouse L cells expressing high levels of the M6P/IGF2R (clone 261-4 #19) were originally obtained from Dr. Peter Lobel, Rutgers University [80]. The differentiated human hepatoma cell line HuH-7 was a gift of Dr. Terrence Donohue of the Department of Internal Medicine at the University of Nebraska Medical Center (UNMC). The human pancreatic adenocarcinoma cell line Capan-1 was obtained originally from the American Type Culture Collection and was a gift of Dr. Surinder Batra of the Department of Biochemistry and Molecular Biology, UNMC. These cell lines were cultured in Dulbecco's modified Eagle medium (DMEM) supplemented with 10% fetal bovine serum, hereafter referred to as complete medium. Human neuroblastoma SK-N-AS cells, obtained from the American Type Culture Collection, were maintained in DMEM supplemented with 10% FBS and 0.1 mM non-essential amino acids. The human pancreatic cancer cell line S2-013 was a gift of Dr. Joyce Solheim, Fred and Pamela Buffett Cancer Center, UNMC. These cells were maintained as an adherent cell line in Roswell Park Memorial Institute (RPMI) 1640 medium supplemented with 10% fetal bovine serum, referred to as complete medium. JEG-3 choriocarcinoma cells, obtained from the American Type Culture Collection, were maintained as an adherent culture in minimum essential medium (MEM) supplemented with 10% bovine growth serum, 2 mM sodium pyruvate, and 0.1 mM non-essential amino acids. All cells were cultured at 37°C in a humidified 5% CO_2_/95% air atmosphere and were sub-cultured as needed using 0.25% trypsin-EDTA. All human cell lines were authenticated by analysis of short tandem repeats by Genetica DNA Laboratories (Burlington, NC), most recently in April 2016.

### Preparation of pentamannosyl 6-monophosphate-derivatized based proteins and peptides

Conjugation of PMP to various proteins and peptides was carried out according to the procedure of Braulke *et al.* as modified previously [27, 81]. Bovine serum albumin (BSA), ovalbumin (OVA), insulin (INS), or the peptides Lys-Tyr-Lys (KYK) or Ser-Tyr-Lys (SYK) were incubated at a concentration of 15 mg/mL in the presence of 0.2 M PMP and 160 mM NaCNBH_3_ at 37°C for 4–5 days as described previously for BSA [27, 82]. The resultant PMP-BSA and PMP-OVA products were dialyzed against 50 mM HEPES, pH 7.4, 150 mM NaCl using Pierce Slide-A-Lyzer® dialysis cassettes G2 (10,000 molecular weight cutoff), then purified by gel filtration on a 30-mL Sephadex G-50 column in phosphate-buffered saline. The flow-through fractions were collected, pooled, and stored at −20°C. The size and overall purity of the PMP-proteins was measured using SDS-PAGE stained with Coomassie blue R250 for protein detection. Successful derivatization of PMP to BSA and ovalbumin was determined by comparing the molecular weight shifts of PMP-modified proteins to lanes containing underivatized protein. The PMP-peptide products were purified on a 30-mL quaternary aminoethyl (QAE)-Sephadex A25 column and eluted by a gradient of 0 to 1 M NaCl in 10 mM HEPES, pH 7.4. Fractions were collected and PMP-derivatized peptides were monitored by absorbance at 280 nm. The size and overall purity of the PMP-peptides was validated using MALDI-TOF mass spectrometry (UNMC Mass Spectrometry and Proteomics Core Facility).

### Analysis of PMP-ligand binding to the M6P/IGF2R

A M6P/IGF2R-Sepharose 4B resin-based radioligand displacement assay was used to evaluate the ability of the PMP-ligands to bind the receptor. Aliquots (20 μL) of receptor resin (50% slurry) were incubated with 1.5 nM ^125^I-PMP-BSA or ^125^I-hGUS (used as tracers) in the presence of increasing concentrations of PMP-based ligands (1 pM to 10 μM) in assay buffer (50 mM HEPES, pH 7.4, 0.15 M NaCl, 0.05% Triton X-100) in a volume of 0.2 mL per tube, for 16 h at 4°C on an end-over-end clinical mixer. As positive and negative controls, parallel assays were done that had increasing concentrations of M6P (0.1 μM to 10 mM) or G6P (1 to 10 mM), respectively. The resin pellets were collected by centrifugation for 1 min at 6,000 × g at 4°C, and were washed with 2 × 1 mL of assay buffer. The tips of the tubes containing the resin pellets were cut and quantified in a WIZARD 1470 Automatic Gamma Counter (PerkinElmer, Inc.). The data were converted into percent binding values based on comparison with the ligand-free controls (designated as 100% radioligand binding). The competitive binding data were graphed as semi-log plots of percent binding vs. concentration of M6P, G6P, or the PMP-ligands. Best-fit curves were generated by nonlinear regression analysis using Prism GraphPad software (San Diego, CA), which also allowed estimation of the IC_50_ value, the concentration of test ligand that displaces 50% of radioligand binding. Values for relative binding affinity (RBA) for the PMP-based ligands were normalized to M6P for a given experiment and are reported as the mean of at least three replicate experiments.

### Expression and analysis of epitope-tagged soluble receptors

Transient expression of soluble receptors was done in HEK 293 cells as described previously [83]. Transfection was carried out by a modification of the calcium phosphate method [84]. At 24 h post-transfection, the medium was replaced with serum-free DMEM followed by incubation for 3 days to permit the cells to condition the medium. To recover the secreted, epitope-tagged soluble receptors, the conditioned medium was recovered and cellular debris was pelleted on a tabletop centrifuge at 7000 RPM. The medium was supplemented with 1 mM PMSF and 1 mM sodium fluoride, and concentrated to ~250 μL using Amicon Ultracel 10K centrifugal filters as per the manufacturer's specifications, and then stored at −20°C until use. For full-length receptors, cells were harvested three days post-transfection and lysates were prepared by solubilization with 50 mM HEPES, pH 7.4, 1% Triton X-100, 1 mM MgCl_2_, 1 mM PMSF, and PIC as previously described [85]. After conditioned media and lysates were prepared, 25 μL aliquots were electrophoresed on 4-20% SDS-PAGE gel under reducing conditions according to the immunoblotting protocol.

### Immunoblot analysis

Whole-cell lysates were resolved by reducing SDS-PAGE and transferred to BA85 nitrocellulose paper (Schleicher & Schuell, Keene, NH). The blots were incubated with blocking buffer (4% non-fat dry milk in 50 mm HEPES, pH 7.6, 150 mm NaCl, 0.1% Tween-20) for 1 h at room temperature, and probed with the appropriate antibody [α-M6P/IGF2R IgG (1:4000 dilution), α-pAkt(Thr308) (1:1000 dilution), α-pAkt(Ser473) (1:1000 dilution), α-Akt (1:1000 dilution), α-pERK (1: 4000 dilution), α-ERK (1:400 dilution), α-β-actin (1:4000 dilution) in blocking buffer] for 1 h at 22°C. A goat anti-mouse or goat anti-rabbit secondary antibody conjugated to horseradish peroxidase was then added for 1 h at RT. The blots were developed using enhanced chemiluminescence (ECL), imaged by exposure to x-ray film, and quantified using Typhoon 9410 PhosphorImager analysis (Amersham Biosciences Corp., Piscataway, NJ) or Li-Cor C-DiGit® Blot Scanner (Li-Cor Biosciences, Lincoln, NE) and Image Studio Lite Software. Alternatively, blots probing for α-FLAG IgG (1:1000 dilution) used a secondary rabbit a-mouse IgG (1:1000). The resulting antibody complex was developed with ^125^I-protein A (7 μCi) and detected by means of autoradiography and quantified using Typhoon 9410 PhosphorImager analysis [34].

### Immunoprecipitation of soluble receptors with M2 α-FLAG affinity resin and competitive binding analysis of PMP-ligands

Aliquots of HEK 293 cell conditioned media and lysates, containing equal amounts of expressed FLAG-tagged soluble receptors as determined by the bicinchoninic acid assay, were incubated with 6 μL of packed M2 affinity resin in HEPES-buffered saline (HBS; 50 mM HEPES, pH 7.4, 0.15 M NaCl) plus 1% BSA and 5 mM M6P with mixing for 16 h at 4°C. The resin was collected by centrifugation at 8,000 × g for 30 s. The resulting resin pellets were washed four times with 1 mL HBS containing 0.05% Triton X-100 (HBST). The immunoprecipitated soluble receptors were incubated with 2 nM ^125^I-PMP-BSA in the presence of increasing concentrations of the PMP-ligands (0 mM, or 1 pM to 10 μM) in assay buffer with mixing for 4 h at 4°C. Resin pellets were processed and the binding data were quantified and calculated as described above for the receptor-resin experiments.

### sIGF2R gel shift analysis

Soluble M6P/IGF2R purified from FBS (sIGF2R 0.4 μM) [86] was incubated at RT for 90 min with concentrations of hGUS or PMP-BSA that approximated a 10:1 molar ratio of ligand to receptor. For native PAGE, samples were mixed with sample buffer (25% sucrose, 250 mM TrisHCl, and 0.05% bromophenol blue, pH 6.8) and run on 4-20% gradient gels (Pierce Precise™ Protein Gels, pH 6.8), as per the manufacturer's specifications with the following modifications; to electrophorese the samples under native conditions, the running buffer was prepared without SDS (125 mM Tris, 96 mM glycine, pH ~8.3). Electrophoresis was carried out at a constant 150V until the tracking dye exited the gel (~45 min to 1 h). Under native conditions, BSA oligomerizes and the additive molecular masses of the oligomerized BSA can be used as approximate molecular weight standards. After electrophoresis, the gels were stained with Coomassie blue and the amount of monomeric sIGF2R remaining in the destained gels was quantified by densitometry using a BioRad Gel Doc with Quantity One 4.5 1-D Software Analysis (BioRad, Hercules, CA).

### Cell growth experiments

To investigate kinetics and dosage of PMP-ligand effects on cultured cells, growth studies were performed using a modification of the previously described MTT vital dye assays [87]. Briefly, cells were seeded into 12- or 96-well plates at 500 to 10,000 viable cells/well, in full serum-containing medium. The cells were incubated for 24 h at 37°C before switching to reduced serum (1% FBS) for acclimation prior to drug treatments. At this point for the time course studies, designated day 0, a set of wells was subjected to the MTT assay while the remaining wells were incubated with the reduced serum supplemented with the various treatments indicated in the figures (vehicle control, M6P, PMP-ligands, or IGF-II) and culture was continued for 1-5 days. 3-(4,5-dimethylthiazol-2-yl)-2,5-diphenyltetrazolium bromide (MTT) at a concentration of 1 mg/mL in phenol red-free medium was added to culture wells for an incubation period of 3 h at 37°C/10% CO_2_. Wells containing purple formazan were solubilized with isopropanol and the absorbance of 100 μL solution was measured using a SpectraMax 190 plate reader (Molecular Devices, Sunnyvale, CA) at 570 nm corrected for background absorbance at 690 nm. The corrected absorbance data have been plotted as a function of time. Adjustments to the aforementioned protocol included HuH-7 and JEG-3 cells cultured in MEM plus 10% bovine growth serum, 2 mM sodium pyruvate, and 0.1 mM non-essential amino acids). For dose-response studies, cells were cultured as previously described with the following modifications: cells were seeded at 3,000 cells/well into 96-well dishes and, after the addition of test compounds, effects of dosage on cell growth were measured after a 48 h incubation at 37°C in a humidified 5% CO_2_/95% air atmosphere.

### Apoptosis studies

Cells were seeded into 24-well plates, and once 70% confluence was reached, the cells were incubated with the treatments in 3% FBS-containing medium. Nuclear staining was done by incubation with 5 μg/mL 2-(4-amidinophenyl)-1H-indole-6-carboxamidine (DAPI) for 20 min at 37°C, 5% CO_2_. Representative images (3-6 images) of the DAPI fluorescence and under phase-contrast illumination were taken on a Leica DMI6000B (Leica Microsystems, Buffalo Grove, IL) fluorescent microscope. The number of apoptotic nuclei, as determined by fragmented and blue staining, and total number of cells were counted, and the percentage of dead cells was determined. To measure caspase 3/7 activity, Capan-1, S2-013, and SK-N-AS cells were seeded into 96-well black, clear bottom plates, similar to the MTT assay. After 24 h, the serum was stepped down for an additional 24 h before treatments. 24 h following the treatments, Apo-ONE® Homogeneous Caspase 3/7 Activity Assay (Promega, Madison, WI) was performed according to manufacturer's instructions. The fluorescence at 499/521 nm was determined by a Tecan Infinite® M200 Pro fluorescent spectrometer (Tecan Systems, Inc, San Jose, CA). For the TUNEL assay, Capan-1 cells were seeded into Nunc^TM^ Lab Tek^TM^ II 8-well chamber slides (Fisher Scientific, Pittsburgh, PA) in complete medium until 70% confluency was reached. Cells were treated for 24 h before staining by DAPI and TUNEL (In Situ Cell Death Detection Kit TMR red; Roche, Basel, Switzerland), according to manufacturer's instructions. Representative fields were captured under fluorescent channels using a fluorescent microscope. TUNEL-positive (indicated by a bright red dot) and total cells (DAPI stained) were counted in a field of 100-150 cells and the percentage of TUNEL-positive cells were calculated.

### IGF-II internalization/degradation assay

An assay was developed to assess degradation and, indirectly, internalization by adding ^125^I-IGF-II to the cells and, after incubation for various times, measuring the integrity of the growth factor by its solubility in trichloroacetic acid (TCA). For these experiments, cells were seeded into 24-well plates. After 24 h, cells were incubated with various treatments or with the vehicle control (HBS + HCl) along with 0.3 nM ^125^I-IGF-II plus 1 nM unlabeled carrier IGF-II in 3% FBS-containing medium. Cells were incubated with 900 μL of medium supplemented with the various treatments and allowed to internalize, degrade and re-secreted the degradation products of the ^125^I-IGF-II for 6, 24, 48, or 72 h. At intervals thereafter, two aliquots (200 μL and 50 μL) were withdrawn from triplicate wells and frozen at −20°C until further use.

Following the incubations and collection of the conditioned media for all time points, precipitation with TCA was performed on the conditioned media to determine the amount of radioactivity that is soluble (representing low-molecular-weight IGF-II degradation products) vs. insoluble/precipitable with acid (intact IGF-II). To the 200 μL aliquots were added 150 μL of 10% BSA (2.3% final concentration) as a bulk carrier co-precipitation prior to addition of 300 μL of 100% TCA (to a final concentration of 46%). Samples were vigorously vortexed and incubated on ice for 1 h. TCA precipitates intact IGF-II (as demonstrated in experiments validating the assay), so the soluble and insoluble fractions were separated via centrifugation at 13,200 rpm, 4°C, 10 min. The supernatant fractions were immediately drawn off and collected in 5 mL gamma counter tubes. The bottoms of the tubes containing the pellets that represent the precipitated, intact IGF-II with carrier protein were cut off and collected into 5 mL gamma counter tubes. Samples were counted on a WIZARD 1470 Automatic Gamma Counter (PerkinElmer Life Sciences) and compared to the total counts per well calculated from counts of the 50-μL aliquots. Each sample from the same well was normalized as a percentage of the total before the mean and the standard error of the mean (SEM) were calculated for each treatment at each time point. To validate the integrity of the input ^125^I-IGF-II in each experiment, the amount of precipitable IGF-II was determined after incubation in cell-free medium for the duration of the assay. Over many experiments, the input IGF-II was found to be ~80% TCA-insoluble when assayed over the 48-72 hours of the experiments, indicating that our radioligand is stable under cell-free conditions (data not shown). We thus interpreted the conversion of radioactive material from TCA-insoluble to TCA-soluble forms to represent the action of the cells on the protein, i.e., internalization and degradation. Additionally, the sum of the recovered counts in the supernatants and pellets was approximately 80-100% of the total counts added to the assay.

### Statistical methods

Comparative analyses among multiple experimental groups were done using a one-way analysis of variance (ANOVA) with Dunnett's test as a post-hoc analysis that compared specific group means (e.g., PMP-ligands, IGF-II, etc.) to a control group mean (medium + vehicle). Differences were considered significant at *P* <0.05 or otherwise noted.

## SUPPLEMENTARY FIGURES


