# Supplementary material for: Inhibition of insulin-like growth factor II (IGF-II)-dependent cell growth by multidentate pentamannosyl 6-phosphate-based ligands targeting the mannose 6-phosphate/IGF-II receptor
Source: Oncotarget. 2016 Aug 22;7(38):62386–410. doi: 10.18632/oncotarget.11493 (PMC5308735; doi:10.18632/oncotarget.11493)
Supplement: Supplementary file 1 [file oncotarget-07-62386-s001.pdf]

Inhibition of insulin-like growth factor II (IGF-II)-dependent cell growth by multidentate pentamannosyl 6-phosphate-based ligands targeting the mannose 6-phosphate/IGF-II receptor

SUPPLEMENTARY FIGURES

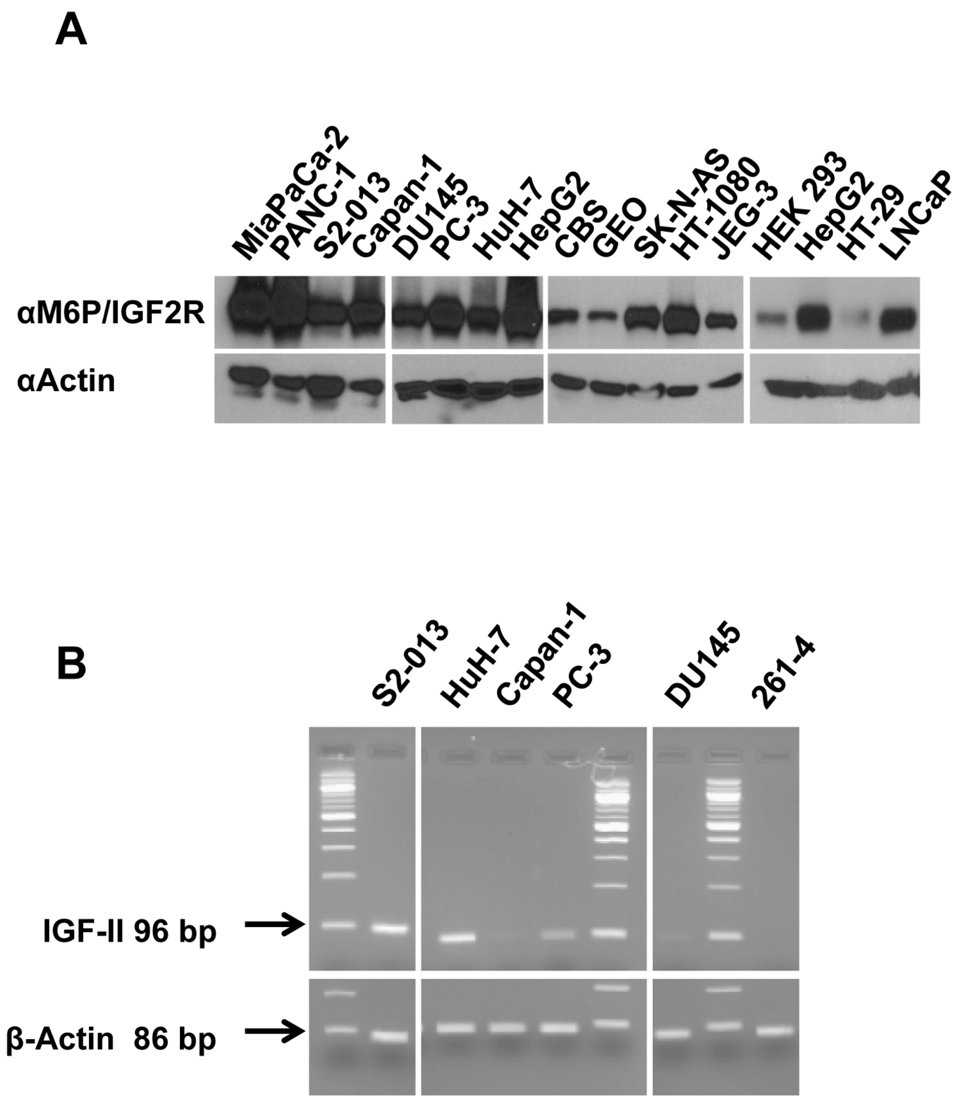

Supplementary Figure S1: M6P/IGF2R and IGF-II expression by cancer cell lines. A. Lysates of various cancer cell lines were subjected to SDS-PAGE and immunoblot analysis, probing with α-CD222 antibody against the M6P/IGF2R. Pancreatic cancer cell lines: MiaPaCa-2, PANC-1, S2-013, Capan-1; prostate cancer cell lines: DU145, PC-3, LNCaP; hepatocellular carcinoma cell lines: HuH-7, HepG2; colon cancer cell lines: CBS, GEO, HT-29; neuroblastoma cell line: SK-N-AS; fibrosarcoma cell line: HT-1080; choriocarcinoma cell line: JEG-3; human embryonic kidney cells: HEK 293. The immunoblots shown represent replicate blots. **B.** RT-PCR analysis of IGF-II expression in various cancer cell lines.

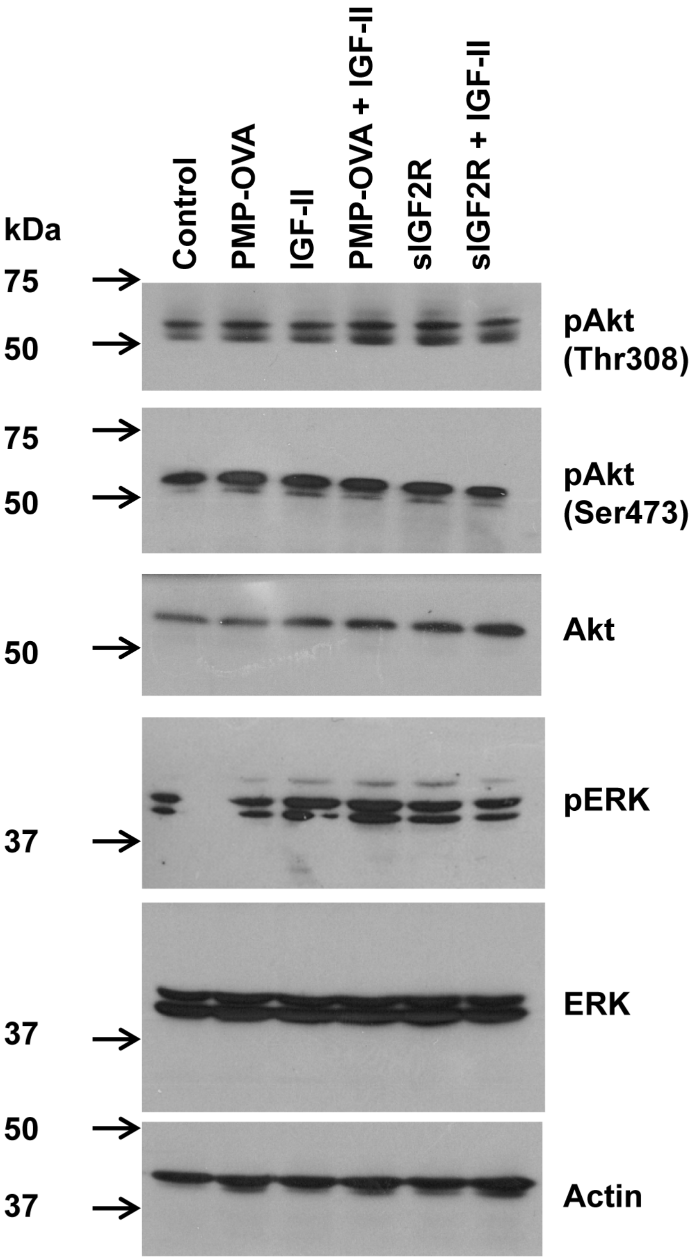

Supplementary Figure S2: Effects of PMP-OVA and IGF-II on mitogenic and survival responses in cancer cell lines. SK-N-AS cells were seeded into 6-cm dishes in complete medium and allowed to attach for 24 h. Cells were treated with IGF-II (10 nM), PMP-OVA (200 nM) ± IGF-II (10 nM), sIGF2R (10 nM) ± IGF-II (10 nM), or vehicle control. Cell lysates were prepared after incubation for 24 h. Aliquots of cell lysate having equal amounts of protein were immunoblotted and probed with the indicated antibodies. The arrows indicate positions of molecular weight marker proteins.
